# How absent negativity relates to affect and motivation: an integrative relief model

**DOI:** 10.3389/fpsyg.2015.00152

**Published:** 2015-03-10

**Authors:** Roland Deutsch, Kevin J. M. Smith, Robert Kordts-Freudinger, Regina Reichardt

**Affiliations:** ^1^Department of Psychology, Technische Universität DresdenDresden, Germany; ^2^Department of Psychology, University of PaderbornPaderborn, Germany; ^3^Department of Education and Psychology, Freie Universität BerlinBerlin, Germany

**Keywords:** relief, avoidance, motivation, reward, appraisal

## Abstract

The present paper concerns the motivational underpinnings and behavioral correlates of the prevention or stopping of negative stimulation – a situation referred to as relief. Relief is of great theoretical and applied interest. Theoretically, it is tied to theories linking affect, emotion, and motivational systems. Importantly, these theories make different predictions regarding the association between relief and motivational systems. Moreover, relief is a prototypical antecedent of counterfactual emotions, which involve specific cognitive processes compared to factual or mere anticipatory emotions. Practically, relief may be an important motivator of addictive and phobic behaviors, self destructive behaviors, and social influence. In the present paper, we will first provide a review of conflicting conceptualizations of relief. We will then present an integrative relief model (IRMO) that aims at resolving existing theoretical conflicts. We then review evidence relevant to distinctive predictions regarding the moderating role of various procedural features of relief situations. We conclude that our integrated model results in a better understanding of existing evidence on the affective and motivational underpinnings of relief, but that further evidence is needed to come to a more comprehensive evaluation of the viability of IRMO.

A fundamental feature differentiating various emotions is whether they refer to present or absent events. For example, the presence of positive events typically triggers happiness, whereas the absence of a desired positive state triggers anger (Carver and Harmon-Jones, 2009). Likewise, the expected presence of negative stimulation (NStim) of all sorts such as pain, social rejection, or failure at work, typically triggers fear, while the prevention or offset of NStim triggers relief (Lohr et al., 2007; Riebe et al., 2012). Generally, many theories of emotion suggest links between emotions, affective valence, and motivational orientations of approach and avoidance, but not so in a consistent manner. Very simplified, one class of theories, which we label *valence theories*, suggests that all positive emotions are associated with approach motivation, whereas all negative emotions are associated with avoidance motivation (e.g., Gray, 1987). A second class of theories, which we label *goal theories*, assumes the key feature along which emotion and motivational orientation are matched to be the goal that is pursued by an actor (e.g., Carver, 2001), whereas motivation and affective valence are seen as orthogonal.

Interestingly, these two classes of theories make markedly diverging predictions when it comes to absence-based emotions such as anger and relief (for discussions, see Carver, 2004, 2009; Carver and Harmon-Jones, 2009). Therefore, studying the affective and motivational underpinnings of absence-based emotions not only improves the understanding of these particular emotions. It also helps evaluating theoretical notions about the relation between emotions and broad motivational systems of approach and avoidance. Carver and Harmon-Jones (2009) recently reviewed existing evidence on the affective valence and motivational orientation associated with anger. Although the relation between anger and motivational orientation seems to be moderated by various factors such as the goal of approach movements (e.g., Krieglmeyer and Deutsch, 2013; Bossuyt et al., 2014), the evidence generally favors the notion that anger is of negative affective valence but derives from an approach motivational orientation. This is in line with core assumptions of goal theories of emotion–motivation interactions (e.g., Carver and Scheier, 1990; Higgins, 2001; Harmon-Jones et al., 2003).

The aim of the present article is to provide an integrative review on the affective and motivational underpinnings of relief, an emotion triggered by the absence of expected or experienced NStim (Lazarus, 1991; Roseman and Evdokas, 2004; Lohr et al., 2007; Leknes et al., 2011; Riebe et al., 2012; Gerber et al., 2014). We are certainly not the first reviewing research on relief (Lohr et al., 2007; Riebe et al., 2012; Bastian et al., 2014; Gerber et al., 2014; Navratilova and Porreca, 2014). Yet, these reviews are focused on theories, paradigms, and findings within a particular range, such as relief from the stopping of pain (Bastian et al., 2014; Gerber et al., 2014; Navratilova and Porreca, 2014), relief from the termination of fear (Lohr et al., 2007; Riebe et al., 2012), long-term decrease in fear responding (Riebe et al., 2012), or neuronal underpinnings of relief (Gerber et al., 2014; Navratilova and Porreca, 2014). Importantly, these reviews were not focused on tackling the questions of affective valence and motivational orientation, and also provide limited cross cutting perspectives. The present review seeks to overcome both limitations. In what follows, we will first briefly explain the importance of relief, and provide a conceptual clarification of relief. We then review diverging theoretical perspectives on the motivational orientation associated with relief and present a first step toward an integrative relief model (IRMO) that aims at combining parts of earlier, more focused theories of relief. We claim that integrating parts of these theoretical fields will help reconciling contradictory conceptualizations of and empirical results on relief. As a derivation, we identify two parameters of relief that can be expected to go hand in hand with differences in valence and motivational orientation. Finally we review evidence on the parameters and discuss the evidence in relation to theoretical notions on relief and motivational orientation.

## THEORETICAL AND PRACTICAL IMPORTANCE OF RELIEF

As suggested above, reviewing affective and motivational underpinnings of relief is important because it helps evaluating diverging theories of emotion–motivation interactions. Gaining a better understanding of relief is also of great importance because relief contributes to a number of phenomena of great practical importance. There is a growing literature on the mechanisms of relief from acute and chronic pain in general (e.g., Leknes et al., 2008, 2013; Bastian et al., 2014), and the role of relief in maintaining self-infliction of harm (e.g., Favazza, 1998; Franklin et al., 2013a). Relief is hypothesized to be a major force in phobia and avoidance behavior (Mowrer, 1960; Lohr et al., 2007). Moreover, although craving positive end-states plays a major role in addiction (e.g., Robinson and Berridge, 1993), there is also a contribution of relief from negative affect (e.g., Baker et al., 2004; Ostafin and Brooks, 2011). Also, relief may promote social influence (Dolinski and Nawrat, 1998; Dolinski et al., 2002) but at the same time may prevent creativity (Baas et al., 2011). Answering the question of whether relief is of positive vs. negative valence, as well as whether it goes along with an approach vs. avoidance motivation will contribute to a better understanding and perhaps ultimately control of these phenomena.

## CONCEPTUAL CLARIFICATION

Emotions are complex constructs involving facets such as subjective experiences, physiological response-patterns, cognitions, and behavioral tendencies that are typically triggered by a class of stimuli. As with other emotions (cf., Ortony and Turner, 1990; Prinz, 2004), formal definitions of which specific manifestations of the above facets constitutes relief slightly differ depending on the author (e.g., Ortony et al., 1988; Lazarus, 1991; Carver, 2001; Roseman and Evdokas, 2004; Lohr et al., 2007; Riebe et al., 2012; Gerber et al., 2014). However, one facet is shared by almost all researchers: relief derives from situations in which an expected or previously experienced NStim is reduced or absent. For example, Roseman and Evdokas (2004, p. 4) characterize relief as a consequence of “...appraising an event as consistent with an aversive pain-minimizing motive...” Lazarus (1991, p. 122) suggests the cause of relief to be “...a distressing, goal-incongruent condition that has changed for the better or gone away.” Leknes et al. (2011, p. 1) characterize relief as “...reward induced through omission or reduction of an aversive event...” Moreover, research in the tradition of appraisal theories has tried to uncover conditions under which people label an affective state as relief. Such studies revealed that appraising negativity as absent was reliably associated with subjective relief (Roseman et al., 1990; Roseman, 1991, 1996), while results for other appraisal dimensions were less clear-cut. In an attempt to capture the essence of existing definitions, the present review will use the term relief to refer to the emotion that is triggered by the absence of expected or previously experienced NStim. Moreover, we will refer to situations in which expected NStim does not occur, or in which experienced NStim stops or is reduced, as relief situations.

## THEORETICAL PERSPECTIVES ON RELIEF

Clearly, relief is part of many emotion theories. As can be derived from **Table 1**, these theories widely agree on the valence of relief, which is identified as positive. There is also agreement that relief presupposes a prior negative situation: “For it is only when an animal anticipates... punishment (fears) that it can be affected by the omission of punishment (‘relief’)” (Gray and McNaughton, 2000, p. 50). But there is some inconsistency in how the omission of experienced vs. expected NStim relates to relief. While the above quote implies that the omission of expected punishment is considered relief, Lohr et al. (2007; cf. Gerber et al., 2014) suggested differentiating between situations where a negative state stops (labeled relief) and situations where the non-occurrence of a potential negative state is experienced (labeled respite), whereas other theorists identify the latter case as relief too. For example, Riebe et al. (2012, p. 164) associate fear relief with recognizing “...the absence and/or disappearance of a threat...” In such situations, the fear associated with threat stops, but the dreaded event was never experienced. Moreover, there seems to be some agreement that relief results from the reduction of the psychological impact of a negative situation. But there is disagreement on whether relief presupposes a certain and complete omission of negativity. Some theories allow for something that Leknes et al. (2013) termed relative relief, where negativity must not necessarily be fully averted but only reduced (cf. Lazarus, 1991; Carver, 2001; Fujiwara et al., 2009; Leknes et al., 2013). Other theories, however, at least implicitly, associate relief with the full and certain omission of negativity (e.g., Roseman and Evdokas, 2004). We believe that all these facets of relief are important and should be considered in an integrated way. In what follows, we will focus on how various theories associate relief with motivational orientations.

### RELIEF, APPROACH, AND AVOIDANCE

As can be derived from **Table 1**, different theories make diverging assumptions about the motivational orientation underlying relief. In line with earlier analyses (e.g., Higgins, 1996, 1997; Carver, 2001, 2009), we recognize two broad clusters of theories relating relief and motivational orientation. *Valence theories* assume affective valence to be the key feature along which emotion and motivational orientation are matched (e.g., Schneirla, 1959; Gray, 1971; Lang et al., 1990, 1992; Neumann et al., 2003; Strack and Deutsch, 2004). For example, Lang et al. (1992, p. 44) suggested “...that pleasant states are driven by the appetitive system and unpleasant states by the aversive motivation system...” Consequently, to the degree that relief can be considered to be of positive valence, relief is assumed to be an emotion of the approach system. Similarly, Gray’s (1987) reinforcement sensitivity theory (RST) states that the valence of stimuli determines whether appetitive [behavioral approach system (BAS)] or aversive motivation [fight–flight system (FFS); behavioral inhibition system (BIS)] dominates behavior. More specifically, the BAS is supposed to be distinctively activated by primary and secondary reward stimuli, including relief, resulting in the formula “hope = relief” (Gray, 1987, p. 248). In essence, valence theories suggest that positive emotions are driven by the approach system, and that therefore relief is an emotion of the approach system.

**Table 1:** Emotion theories and their assumptions regarding the association between relief and valence, as well as approach and avoidance motivation.

Name of theory	Central publication	Origin of relief	Valence	Motivational orientation
Reinforcement sensitivity theory	Gray (1971)	Stimuli that predict avoidance of aversive stimulus activate behavioral approach system	Positive	Approach (activation)
Revised reinforcement sensitivity theory	Gray and McNaughton (2000)	Stimuli that predict avoidance of aversive stimulus activate behavioral approach system Anticipation of alternative outcomes may activate behavioral inhibition system	Positive	Approach (activation) Approach (deactivation)
Emotional reflex theory	Lang et al. (1990, 1992)	No specific notion, but pleasant states are “driven” by approach system	No statement	Unclear (approach if relief assumed as positive)
Opponent process theory	Solomon (1980)	NStim triggers *A* process, which activates counter-regulatory *B* process. If *A* process stops, *B* process prevails due to slower build-up and slower decay	Positive	Depends on quality of A process. Approach for relief from fear or pain.
Self-regulation theory	Carver and Scheier (1998)	Rate of progress toward the attainment of an avoidance goal exceeds the criterion rate of progress	Positive	Avoidance (activation and deactivation)
Regulatory focus theory	Higgins (1997)	Successful pursuit of a prevention (i.e., avoidance) goal	Positive	Avoidance
Cognitive-motivational-relational theory of emotions	Lazarus (1991)	Shift from appraising a situation as goal incongruent (i.e., undesirable) to goal congruent (i.e., desirable)	Positive	Unclear but general deactivation
OCC model	Ortony et al. (1988)	Disconfirmation of negative expectations	Positive	Unclear
Emotion systems model	Roseman (1984, 2013)	Appraisal that a situation is consistent with the motive to avoid punishment	Positive	Avoidance (deactivation)
Belief-desire theory of emotions	Reisenzein (2009)	Disconfirmation of a prior belief that an undesired state of affairs is the case	Positive	Not stated

*Goal theories* assume the key feature along which emotion and motivational orientation are matched to be the type of goal that is pursued by an actor (e.g., Higgins, 1996, 1997; Carver and Scheier, 1998; Carver, 2001). Whereas valence theories assume that all positive affects (e.g., elation and enthusiasm) are associated with approach motivation and negative affects (e.g., fear and distress) with avoidance motivation, goal theories assume that valence is orthogonal to approach/avoidance. Rather, valence is hypothesized to be strongly dependent on the success of the goal pursuit (Higgins et al., 1997; Carver and Scheier, 2011). Accordingly, positive as well as negative affect can result both from approach and avoidance motivation. If an avoidance goal is pursued, doing poorly is predicted to result in anxiety and fear, whereas doing well will result in relief and calmness (Carver, 2001). Therefore, goal theories suggest relief to be a positive affect that derives from avoidance processes. Importantly, some goal theories explicitly suggest that relief derives from avoidance motivation but at the same time deactivates avoidance motivation (e.g., Roseman, 2013). Other goal-theories are less clear about whether relief activates or deactivates avoidance motivation. Carver’s (2001) theory suggests that emotions provide feedback on the success of goal pursuit, with relief signaling that avoidance processes are progressing well. This suggests that relief might occur even when the avoidance goal is not yet fulfilled. From this perspective, assuming relief to deactivate avoidance processes would be dysfunctional. At the same time, the theory suggests that relief is “...part of the process... of regrouping, restoring one’s access to energy supplies... preparatory to turning to some new activity” (Carver, 2001, p. 351), which may imply abandoning avoidance goals.

A third theory ascribes a dual motivational nature to relief. Specifically, the revised version of Gray’s RST (Gray and McNaughton, 2000) maintains the notion that relief situations activate the BAS. However, the theory also suggests that in relief situations “...both the behavioral inhibition and the BAS will be activated concurrently, with some patterns of behavior being produced by the one system and some by the other” (Gray and McNaughton, 2000, p. 55). One reason for this prediction is that stimuli associated with (successful) avoidance behavior “...can, and often will, predict that some other (usually many other) responses will produce, or fail to avoid, the aversive stimulus” (Gray and McNaughton, 2000, p. 55).

**FIGURE 1:**
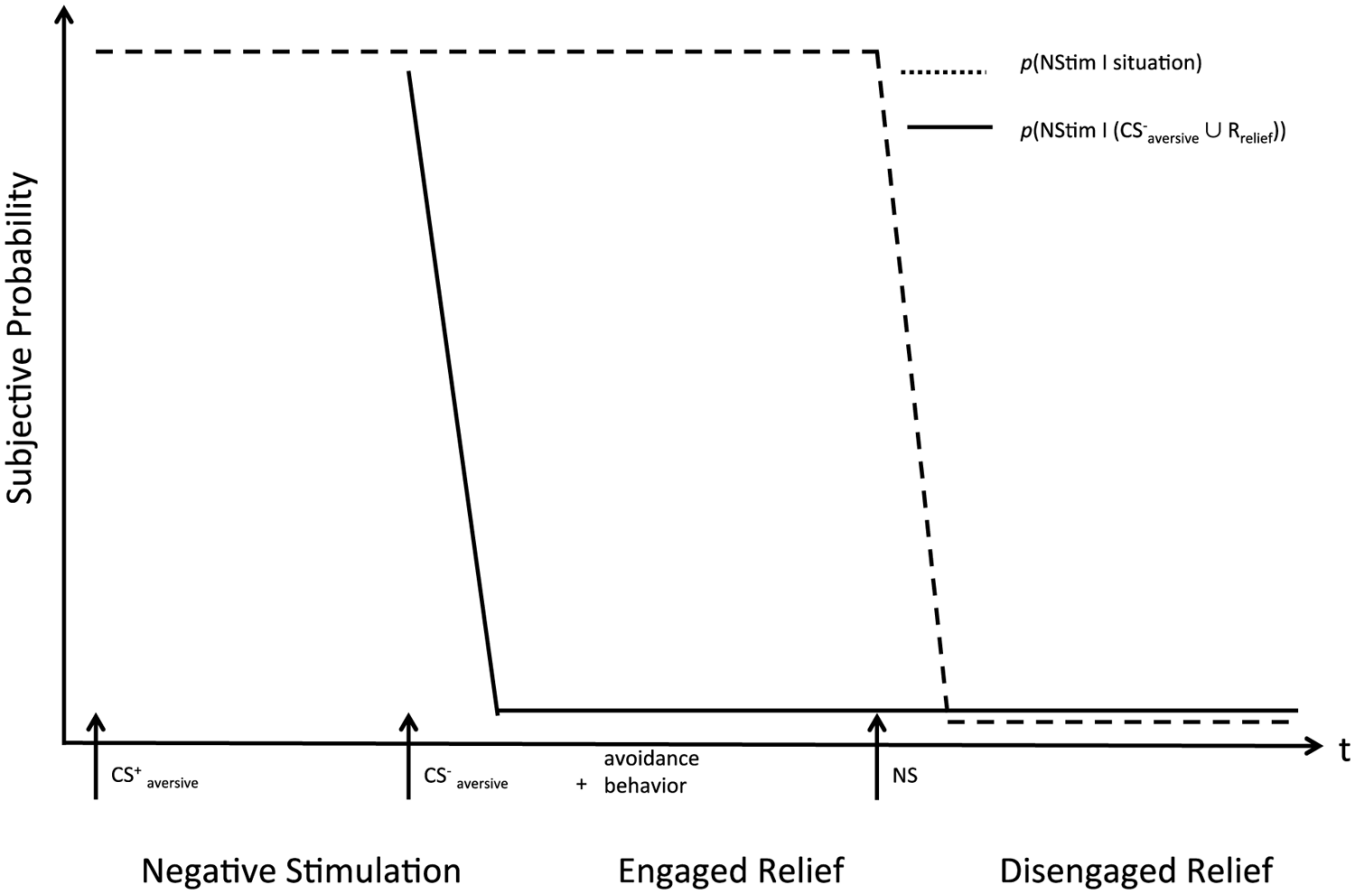
**Idealized phases of relief along with exemplary stimuli.** CS^+^_aversive_: a threat stimulus signaling the onset of punishment. Avoidance behavior: behavior effective in preventing punishment (i.e., counteracting the CS^+^_aversive_). NS: a neutral context where punishment is unconditionally absent. During engaged relief, threat is absent on the condition that avoidance behavior is executed, otherwise it is present. During negative stimulation, threat is unconditionally present, during disengaged relief, threat is unconditionally absent.

A final theory has been immensely influential on relief researchers (e.g., Leknes et al., 2008; Andreatta et al., 2013), but makes only conditional predictions regarding the motivational orientation of relief: opponent process theory (OPT; Solomon, 1980). OPT suggests that a psychophysical process *A* typically triggers a process *B* that counteracts the effect of the original process *A*. Moreover, “The *B* process (the opponent process) is postulated to be (a) of sluggish latency, (b) inertial, or slow to build to its asymptote, and (c) slow to decay...” (Solomon, 1980, p. 699). In the case of pain (or other intense aversive stimulation), the *B* process is predicted to be of positive valence and “... individuals feel an emotional state which entails opponent, namely appetitive properties” (Andreatta et al., 2013, p. 1). OPT therefore conceptualizes relief as the persisting *B* process after NStim (Leknes et al., 2008). OPT’s predictions for affective valence are straightforward: “Because the *b* process is an opponent process, its affective or hedonic quality must be opposite to that of the *a* process” (Solomon, 1980, p. 699). But what is the opposite motivational orientation of an *A* process representing unspecific negative affect (Russell, 2003) or specific negative emotions such as fear, anger, or sadness, from which one might feel relieved if they stopped? To answer this question, one must obviously know the motivational orientation associated with the *A* process. As explained in the previous paragraphs, however, this is still a question of considerable debate. For example, from the perspective of goal theories (Carver and Harmon-Jones, 2009), anger belongs to the approach system, so that the opposite motivational orientation would be avoidance. From the perspective of valence theories (Lang et al., 1992), anger belongs to the avoidance system, so that the opposite motivational orientation would be approach. Clearly, OPT makes easy predictions as long as affect is concerned. Moreover, fear or pain as *A* processes go along with avoidance motivation in all considered theories, and hence OPT predicts relief to be approach-oriented in these cases. But predictions regarding other emotions or hedonic states, such as hunger, require additional theoretical assumptions regarding the relations between these constructs.

**FIGURE 2:**
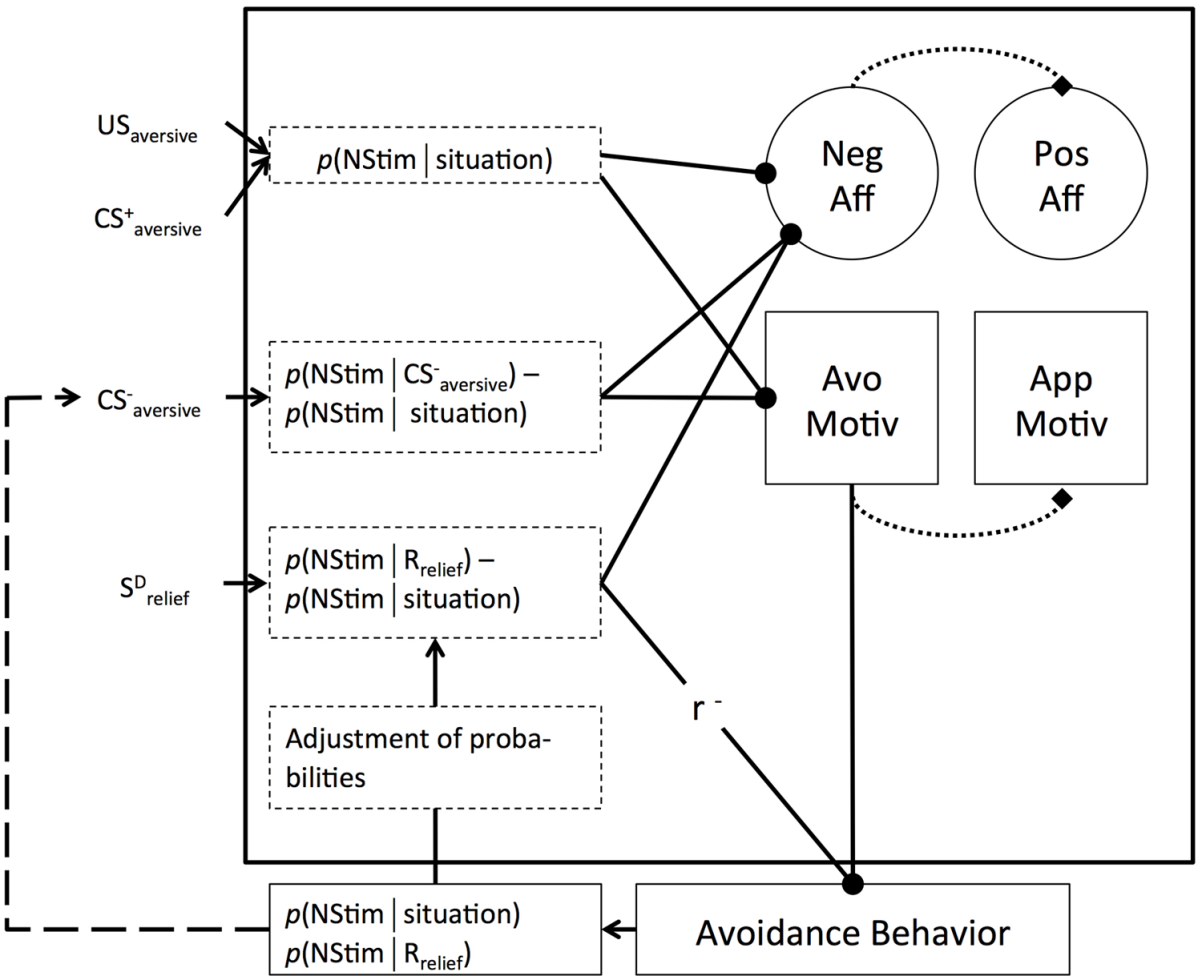
**Overview over core processes of IRMO. -∙** inhibitory if input has negative sign; excitatory if input has positive sign; r^-^ reversal of sign of input; → information flow; dotted boxes represent appraisal and comparison processes; dotted lines represent activation in the sense of OPT. Broken line represents optional path in which action outcome serves as CS^-^_aversive_ (see text for further explanation).

### TOWARD AN INTEGRATIVE MODEL OF RELIEF

Given the heterogeneity of theoretical assumptions on the concept of relief and its affective and motivational bases, we suggest that integrating these diverging views is a pressing goal. In what follows, we describe a first version of an IRMO. While construing IRMO, we draw on four classes of existing emotion theories relevant to relief: theories of fear and learning (Lang et al., 1990; Gray and McNaughton, 2000; Lohr et al., 2007) provide a taxonomy of different relief situations and cues that feed into relief. We included assumptions of regulatory theories of emotion (Carver and Scheier, 1990, 2002; Carver, 2001) regarding the dynamic nature of relief and the feedback function of positive affect during active relief. We draw on mechanisms of OPT (Solomon, 1980; Leknes et al., 2008; Andreatta et al., 2010) to explain the occurrence of positive affect as a consequence of the absence of expected or experienced NStim. Finally, we incorporate basic notions of appraisal theories (Lazarus, 1991; Roseman and Evdokas, 2004; Reisenzein, 2009), highlighting the importance of certainty- and motive-congruence appraisals. Also, IRMO specifically combines assumptions of goal theories of motivation and affect (Higgins, 1996; Carver, 2001) with assumptions of OPT (Solomon, 1980). As suggested by goal theories, IRMO assumes relative independence of the generation of positive vs. negative affect on one hand, and the instigation of approach vs. avoidance motivation on the other hand. Applying OPT, IRMO assumes that both affect and motivation come with their sets of specific *A* and *B* processes, with both following the principles outlined in OPT. Hence, the parts of which IRMO is made are not new, but we consider their combination an innovative step forward toward a better understanding of psychological processes related to absent negativity.

### DYNAMIC NATURE

In line with regulatory theories of emotions (e.g., Higgins, 1996; Carver, 2001), we suggest that relief is best understood as part of a dynamic process instead of a static, one-shot phenomenon. First, relief is a dynamic phenomenon because it presupposes a change from expected or experienced NStim toward their reduction or full absence. Second, relief is a dynamic phenomenon because the shift toward full absence often may evolve over a longer action sequence during which the intensity of positive affect signals the effectiveness of avoidance behavior (Carver and Scheier, 1998; Lawrence et al., 2002). IRMO therefore suggests a tri-phasic sequence of relief episodes, which is an idealized abstraction of a continuous progression from unconditional NStim to unconditional absence of NStim (see **Figure 1**). IRMO suggests that organisms monitor two probabilities throughout these phases, and each phase is characterized by a specific combination of these two probabilities. The subjective probabilities will correlate with objective probabilities but are subject to biases associated with probability estimation (e.g., Gilovich et al., 2002). The first is the probability of NStim in the current situation without any salient features related to safety, expressed as *p*(NStim|situation). The situation may include cues that correlate with the occurrence of NStim (CS^+^_aversive_; e.g., the smell of a dentist’s office), or the actual exposition to aversive stimuli (US_aversive_; e.g., the drill touching the dental pulp). In the latter case, *p*(NStim|situation) necessarily equals 1. The second is the probability of NStim if cues related to safety become salient in the situation. Such cues are safety signals (CS^-^_aversive_; e.g., the sound of an air-conditioning system that just sprang into action), or avoidance behaviors (R_relief_; e.g., running away from a fire). We express this as *p*(NStim|(CS^-^_aversive_ ∪ R_relief_)) ^1^. The availability of R_relief_ can be signaled by discriminative relief stimuli (S^D^_relief_; e.g., the sight of a box of aspirin signals that taking aspirin will stop pain), and therefore perceiving S^D^_relief_ will result in a decrease in *p*(NStim|R_relief_).

The *negative stimulation phase* (NStim phase) is characterized by the experience or expectation of NStim, with *p*(NStim|situation) = 1 in the case of experience, and *p*(NStim|situation) > fear threshold in the case of expectation. *Fear threshold* is the probability at which an individual starts experiencing fear in the face of a threat. It may vary as a function of type of the potential NStim and as an individual difference factor. In the NStim phase, no R_relief_ are available, going along with appraisals of low controllability, although organisms will likely start searching for available R_relief_. Also, no CS^-^_aversive_ or S^D^_relief_ are present that might result in a decrease in the subjective probability of NStim.

The *engaged relief phase* is characterized by the shift from a subjectively high absolute probability of NStim toward a lower absolute probability assessment. Processing CS^-^_aversive_, S^D^_relief_, or engaging in R_relief_ is responsible for the subjective change in probability. In the engaged relief phase, the original threat is still present, such that there is a high probability of NStim if nothing is done and no safety cues are present. There is, however, a lower *p*(NStim|(CS^-^_aversive_ ∪ R_relief_)), that is a lower probability of NStim if avoidance behavior occurs and/or safety signals are present.

The final *disengaged phase* is characterized by the mental disengagement from earlier punishment, threat, safety cues, and avoidance behavior. In this phase, *p*(NStim|situation), and *p*(NStim|(CS^-^_aversive_ ∪ R_relief_)) are appraised as equally low and below the fear threshold. Therefore, this phase is characterized by unconditional safety.

### PROCESS ASSUMPTIONS

#### Negative stimulation phase

The integrative relief model proposes a cascade of processes mediating the shift from NStim to disengaged relief (see **Figure 2**). Starting point is the appraisal of a situation or a concrete stimulus (US_aversive_ or CS^+^_aversive_) in that situation, resulting in the generation of a subjective *p*(NStim|situation) > fear threshold. Such negative appraisals go along with the activation of negative affect and avoidance motivation. Both are considered as *A* processes as conceptualized in OPT, and hence they are assumed to trigger opponent *B* processes, characterized by a slower temporal dynamic and lower intensity than the *A* processes (Solomon, 1980; Leknes et al., 2008). In response to an initial negative appraisal and resulting negative affect, a search for potential coping opportunities will set in. This includes scanning the environment for CS^-^_aversive_ or S^D^_relief_ and scanning memory for avoidance schemata (R_relief_ ) that fit the current situation. As long as this search is without success, the organism remains in the NStim phase.

#### Engaged relief phase

If a safety signal is detected, *p*(NStim|CS^-^_aversive_) will be estimated and compared to *p*(NStim|situation). The intensity of the affectively negative *A* process as well as the motivationally avoidant *A* process will be reduced to the degree that the safety signal is appraised as reducing the probability of NStim ^2^. Based on the principles of OPT, the reduction in the negative affective and motivationally avoidant *A* processes will result in a temporary relative strengthening of the positive affective and approach-oriented *B* processes. In other words, recognizing an increase in safety signaled by environmental stimuli goes along with a positive affective signal and a shift away from avoidance and a relative strengthening of approach motivation generated on the basis of opponent processes.

If an S^D^_relief_ is detected, an additional regulation loop is expected to set in (Carver and Scheier, 1990; Carver, 2001; Lawrence et al., 2002) that serves the specific affordances of active avoidance. An S^D^_relief_ goes along with high controllability appraisals, which are expressed by the difference in the probability of NStim with avoidance behavior and without avoidance behavior [i.e., *p*(NStim|R_relief_) – *p*(NStim|situation)]. Perceiving controllability will result in a decrease in the negative affective *A* processes and hence a temporary upswing in positive affect based on opponent processes. It will further result in an activation of the avoidance behavior that was the basis of the controllability appraisal ^3^. Importantly, IRMO does not predict that perceiving S^D^_relief_, appraisals of controllability, or avoidance behavior directly decrease avoidance motivation. This prediction is based on the notion that avoidance motivation is the energizing part of avoidance behavior. As such, a successful organism will maintain high avoidance motivation as long as avoidance behavior is necessary to generate relative safety.

The actual *p*(NStim|situation) and *p*(NStim|R_relief_) are monitored, and the originally expected probabilities are adjusted based on the observed ones. If the adjustment results in an increase in expected controllability (i.e., the probability difference becomes more negative), the inhibition of negative affect will further increase, resulting in a temporary increase in positive affect based on OPT. Moreover, the avoidance behavior will be further activated. If the observed controllability is worse than the expected one, the inhibition of negative affect will be reduced, going along with a decrease in the activation of the avoidance behavior. If the R_relief_ increased the *p*(NStim) compared to doing nothing, negative affect will increase, going along with an inhibition of the avoidance behavior. This provides a feedback loop driven by the tracked success of ongoing avoidance behavior (Carver and Scheier, 1990; Carver, 2001; Lawrence et al., 2002), potentially resulting in a situation with high controllability appraisals, phases of dominant positive affect (if controllability improves), and high avoidance motivation maintained by the continuously perceived threat of *p*(NStim|situation).

In some cases, instrumental behaviors may generate outcomes that signal the absence of threat for a distinct period of time (Berger and Brush, 1975; Berger and Starzec, 1988). That is, behavioral outcomes may function as CS^-^_aversive_, and IRMO predicts such signals to reduce avoidance motivation and consequently avoidance behavior as long as they are present (see broken line in **Figure 2**). For two reasons, such self-generated CS^-^_aversive_ can even be expected to have more intense effects than external CS^-^_aversive_ (cf. Cándido et al., 2004). First, because they are part of the instrumental action, they may receive more attention than stimuli that are only passively observed, resulting in better learning (e.g., Hommel, 2010). Second, probability estimates of self-generated CS^-^_aversive_ may be more optimistic than passively acquired ones. Contingency assessments are biased toward overestimating control (Langer, 1975), especially under conditions of acting (Langer and Roth, 1975; Blanco et al., 2011). For the same reasons, active relief in general may be more positive than passive relief even when no safety period is signalled (cf. Eder and Dignath, 2014).

#### Disengaged relief phase

According to IRMO, a shift toward disengaged relief goes along with reductions in *p*(NStim|situation), indicating that the situational threat is eliminated. Situational threat can be appraised in multiple ways. One way is to briefly stop avoidance behavior and explore the results. For example, a person who has been taking a pain reliever may briefly stop doing so to see whether the pain is still there. Another way would be to check whether an obvious cause of the threat is gone (e.g., whether the dangerous stray dog has been captured). The principles outlined in IRMO (see **Figure 2**) imply that if *p*(NStim|situation) is reduced, the activation of negative affect and avoidance motivation *A* processes is reduced, and approaches zero if situational threat falls below the fear threshold (cf., Pekrun et al., 2002; Roseman, 2013). As a consequence, avoidance behavior will lose momentum and *B* processes (positive affect and approach motivation) will dominate for a while. As has been theorized by Carver (2009, p. 133), relief “...represents a signal that the person does not have to attend to the threat any longer and attention broadens to consider other available possibilities for goal pursuit...” This, according to Carver (2009), only applies when the threat is eliminated. In that sense, a shift toward new, attractive goals can be expected primarily for the disengaged relief phase.

### APPRAISALS

What is the relationship between the processes specified in the IRMO and appraisals proposed by appraisal theories of emotions (e.g., Roseman, 1984; Ortony et al., 1988; Lazarus, 1991)? We argue that some of these processes can be conceptualized as appraisals. In the case of active relief, we argue that the expected reduction in the probability of NStim through avoidance behavior can be conceptualized as an appraisal of controllability (cf. Mowrer, 1960). Moreover, assessing the absolute probability of NStim as low, or perceiving a reduction in the probability of NStim can be seen as an appraisal of motive congruency – i.e., with the motive to avoid or end punishment (Roseman, 2013). More generally, all probability assessments of IRMO can be thought of as appraisals of certainty of the respective events (i.e., absence or presence of NStim). These appraisals determine the strength of the activating or inhibiting effect on the aversive *A* process.

### CONSEQUENCES FOR THE RELATION TO AFFECT AND MOTIVATION

There are numerous empirical consequences that follow from these process considerations. IRMO was designed to incorporate as many known relief phenomena with as few process assumptions as possible. So it comes as no surprise that it indeed covers many of these phenomena. But many of these consequences are not specific to the question whether relief is associated with approach vs. avoidance motivation and positive vs. negative affect, which is why we will refrain from discussing them here. In addition, the goal of IRMO is to provide an integrative perspective on the affective and motivational underpinnings of relief, which we found to be conceptualized quite differently in various theories. In what follows, we will describe how the principles outlined in IRMO might help to reconcile these perspectives. Particularly, we will discuss procedurally different forms of relief (prevention vs. stopping-relief; active vs. passive relief; see **Table 2**) and demonstrate that IRMO predicts them to relate to approach and avoidance motivation to varying degrees. Although the crossing of the two features (active vs. passive; prevention vs. stopping) suggests four types of relief, we will discuss empirical consequences in the sense of main effects of the two features.

The dynamic perspective of IRMO suggests that affective and motivational underpinnings of relief coarsely vary depending on the relief phase. In the NStim phase, negative affect and avoidance motivation prevail, while in the disengaged relief phase, positive affect and approach motivation prevail. In the engaged phase, the affective tone is positive but motivational orientation differs depending on whether relief is active (avoidance) or passive (approach). Importantly, the phases are characterized by different combinations of subjective probabilities, corresponding to certainty appraisals of predictions regarding NStim or its absence. As a consequence, the certainty of these predictions can be considered moderator variables that affect the intensity and quality of the processes outlined in our model.

**Table 2:** Forms of relief in IRMO.

Prevention stopping	Active passive	Example
Stopping	Passive	Experienced painful stimulation simply ends
Stopping	Active	Experienced painful stimulation is ended through own behavior
Prevention	Passive	A stimulus signals that a feared event will not occur
Prevention	Active	Behavior is executed through which feared negative event will be avoided

### CERTAINTY

The integrative relief model predicts the certainty of NStim to positively correlate with negative affect and avoidance motivation (cf. Ortony et al., 1988), and, in line with learning and appraisal theories, also to positively correlate with positive affect and approach motivation if NStim is prevented or stopped (cf. Ortony et al., 1988; Gray and McNaughton, 2000; Reisenzein, 2009). IRMO further suggests that the certainty with which NStim can be avoided positively correlates with positive affect (cf. Roseman, 1984; Ortony et al., 1988), whereas it’s relation to motivation depends on whether relief is active (no effect) or passive (decrease in avoidance). Depending on the certainty of expected non-punishment compared to expected punishment, IRMO predicts fear to be reduced in intensity and opponent processes of fear to dominate. Hence, the certainty of absence of NStim determines the relative strength of positive vs. negative representations and approach vs. avoidance motivation at a given point in time. This is at odds with a view of relief as a purely positive emotion, but, as will be seen later, backed up by evidence.

### ACTIVE vs. PASSIVE

A first focal feature that differentiates theories, research, and findings in the realm of relief is whether the relief is caused by behavior of the subject or whether the relief occurs independently from the subject’s behavior. Based on an earlier analysis by Zvolensky et al. (2000), Lohr et al. (2007) provide a taxonomy for the realm of anxiety disorders that we deem to also be highly useful outside the clinical context. They suggest differentiating between offset control of aversive stimulation on one hand, and offset prediction on the other hand. Offset prediction is a prototypical example of passive relief, as in Pavlovian conditioning, where CS^-^_aversive_ elicit relief from fear of NStim (Gray, 1971; Cole and Miller, 1999; Genud-Gabai et al., 2013). Offset control of NStim is a prototypical example of active relief, where overt behaviors of a subject cause the prevention or the stopping of a negative event.

#### Engaged relief phase

The integrative relief model assumes active vs. passive relief to correspond to differences in underlying processes for engaged relief. Active relief presupposes engaging in behaviors that cause aversive stimulation to stop or to be prevented, whereas passive relief does not. According to the assumptions made in IRMO, affect and motivation respond in a manner that distinctively supports passive or active relief. If a CS^-^_aversive_ is processed, the expectancy of NStim drops and negative affect and avoidance motivation *A* processes are reduced accordingly. As a consequence, a temporary increase in positive affect and approach motivation will result. Active avoidance behavior will become less likely. Instead, the safety phase signaled by the CS^-^_aversive_ goes along with a higher probability of approach behaviors to be triggered by environmental cues.

For active relief, reducing avoidance motivation as a consequence of processing an S^D^_relief_ would be dysfunctional. S^D^_relief_ signal the opportunity to actively reduce the probability of NStim based on a comparison of the probability of NStim under the condition of action vs. inaction. If this comparison results in an appraisal of controllability, negative affect will be reduced, temporarily resulting in an overshoot of positive affect *B* processes. Avoidance motivation, however, is not reduced. Instead, the appropriate avoidance behavior is activated, energized by avoidance motivation, and its success is monitored. If the behavior reduces the probability of NStim as expected, affect and activation of the behavior remain the same. If the reduction in the probability of NStim is greater than expected, negative affect will further be reduced and another temporary positive affect *B* process will emerge and the avoidance behavior is further activated. If the reduction in the probability of NStim is smaller than expected, activation of negative affect will increase and the avoidance behavior will be inhibited (Carver, 2001, p. 20; Lawrence et al., 2002). If active relief involves the generation of CS^-^_aversive_ that signal the absence of threat for a period of time, active and passive relief will be indistinguishable during the safety period predicted by the behavior-generated CS^-^_aversive_. Taken together, this suggests that in the engaged phase, active relief goes hand in hand with temporary positive affect and avoidance motivation, whereas passive relief goes hand in hand with temporary positive affect and a shift toward approach motivation. As argued above, the effects on valence may be more pronounced for active than for passive relief. A special case are signaled safety periods in active avoidance, which are predicted to resemble passive relief but may exert stronger effects due to heightened attention and illusions of control (see process assumptions; Hommel, 2010; Blanco et al., 2011).

#### Disengaged relief phase

Once *p*(NStim|situation) falls below the fear threshold, the shift toward the disengaged phase has occurred. The process differences in the engaged relief phase extend their effects on affect and motivation to the disengaged phase. Generally, if the situational threat is eliminated at the beginning of disengagement, this goes along with a reduction in the activation of avoidance motivation, which would then allow *B* processes of approach motivation to dominate for a while. Passive relief in the engaged phase, however, already goes hand in hand with a reduction of negative affect and avoidance motivation, such that *B* processes already dominate during engagement. Therefore, at disengagement, passive relief will generate a weaker overshoot of *B* processes than active relief. In the latter case, avoidance motivation was in full activation during engagement, and consequently *B* processes too. If avoidance sets off at disengagement, *B* processes are still active at a high level.

### PREVENTING vs. STOPPING

Theories, research procedures, and observations greatly differ with respect to whether the absence of negativity comes in the form of preventing or in the form of stopping NStim. IRMO adopts parts of Lohr et al.’s (2007) theoretical reasoning in assuming that prevention relief (i.e., an expected NStim does not materialize) and stopping relief (i.e., NStim is experienced but then ends) are associated with different processes. This is also in line with Gray and McNaughton (2000, p. 52) who concluded: “... we need to distinguish carefully between the primary events (Pun^+^, Rew^-^), on the one hand, and CSs for those primary events, on the other, since they can have quite opposite eliciting properties and functional requirements”. As we will see, IRMO generates diverging predictions for affect and motivation as a function of prevention vs. stopping.

#### Negative stimulation phase

In the NStim phase (see **Figure 1**), prevention relief implies that the person has generated an expectation of NStim, be it based on the context or the presence of a CS^+^_aversive_. This requires anticipation processes based on learned associations, as well as appraisal processes that infer motive incongruence, and varying certainty depending on the predictive validity of the CS^+^_aversive_. Also, NStim is still appraised as uncontrollable through own behavior, although a search for such control-opportunities might set in. As a result, fear of NStim will be experienced (cf. Riebe et al., 2012), and, in line with the assumptions of OPT (Solomon, 1980; Leknes et al., 2008), processes opposing fear will set in. In the case of stopping relief, the experience (instead of the anticipation) of NStim (US_aversive_ in **Figure 2**) represents the foundation of the NStim phase. Depending on the type of NStim (e.g., food deprivation, noise, tissue damage), different sensations and emotions (e.g., hunger, frustration, pain) will result. Appraisals include motive incongruence, high certainty and low controllability of NStim. Also, opponent processes specific to the quality of the NStim will set in. In the NStim phase, prevention relief therefore differs from stopping relief in that it presupposes anticipation processes, and involves mainly fear and varying certainty, whereas stopping relief involves no anticipation, diverse negative sensations and emotions, and high certainty appraisals. However, in this phase, both forms of relief go along with negative affect and avoidance motivation.

#### Engaged relief phase

In the case of prevention relief, CS^-^_aversive_ or S^D^_relief_ signal that a previously expected NStim will not occur. The comparison between past (*p*(NStim|situation)) and present (*p*(NStim|(CS^-^_aversive_ ∪ R_relief_)) expectancies of NStim will yield a reduction in fear that is proportional to the drop in expectancies. That is, prevention relief corresponds to a reduction or stopping of fear of US_aversive_ (cf. Riebe et al., 2012). Stopping relief, however, is based on the actual experience of a US_aversive_, which then stops. For this to occur, no expectations and no previous experience with the stimulus or the general situation are necessary – the NStim may simply end (cf. Leknes et al., 2008). The comparison process between past and present experience of US_aversive_ will yield a drop in negative affect proportional to the drop in US_aversive_.

There are three important predictions derived from the process-differences outlined above. First, prevention relief comes, on average, with a greater degree of uncertainty than stopping relief. This follows from the notion that in the case of stopping relief, the desired end-state (i.e., the reduction or stopping of NStim) is experienced and thus factual, whereas in the case of prevention relief, the desired end-state is only detected based on counterfactual reasoning (i.e., the observation that the expected NStim does not materialize). Detecting the validity of a CS^-^_aversive_ presupposes a highly accurate representation of the typical timing of US_aversive_. For example, in order to be certain that a local anesthetic at the dentist’s office really prevents pain, knowledge about when exactly pain can be expected during the treatment is necessary. If such knowledge is absent or imprecise, a residual fear that the CS^-^_aversive_ did not work may prevail until the treatment is over. Detecting the offset of a US_aversive_, on the other hand, is clearly perceivable. For example, if a dentist applies a local anesthetic to stop a toothache, this will result in a drop in pain that in itself is 100% certain. As a consequence, stopping relief as compared to prevention relief will be associated with stronger inhibition of negative affect and stronger corresponding positive affect resulting from *B* processes. Likewise, a stronger decrease in avoidance motivation and a stronger corresponding increase in approach motivation can be expected for stopping compared to prevention relief as long as it is not active (see previous section).

Second, the differences in certainty and perceivability of prevention vs. stopping relief can also be expected to correspond to a difference in the speed of change in subjective probabilities of NStim. More specifically, the offset of NStim will correspond to a sudden decrease in the subjective probability of NStim, whereas the uncertainty that goes along with the ambiguity of the negation of an expectation in prevention relief will result in a more gradual change in the subjective probability of NStim. OPT mechanisms suggest that sudden decreases in negative *A* processes result in a stronger dominance of positive *B* processes than gradual decreases, and data strongly support this conclusion (Leknes et al., 2008). Thus, stopping relief can be expected to result in a more sudden offset of negative affective *A* processes and therefore a stronger overshoot in positive affective *B* processes than prevention relief. For the same reason, a stronger overshoot of approach motivation in the case of stopping instead of prevention relief can be expected, but only if it was not active (see previous section).

Third, elicited counter-regulatory processes as described in OPT can be expected to differ profoundly for prevention vs. stopping relief. In the case of stopping relief, the person has endured *A* processes related to factual NStim (e.g., pain, shame) for some time. Consequently, *B* processes specific to this NStim (e.g., activation of the endogenous opioid system in the case of pain; Leknes et al., 2008, p. 800), and the quality of these processes feeds into the experience of relief after the reduction or stopping of NStim. In the case of prevention relief, the feared NStim (e.g., pain) is not immediately experienced prior to a CS^-^_aversive_ or prior to avoidance behavior. Instead, it is rather the fear of the NStim (e.g., fear of pain) that constitutes the *A* process, and therefore opponent processes to fear act as the *B* process that uniformly shapes prevention relief. In other words, this reasoning results in the prediction that stopping relief will be psychologically and physiologically quite diverse depending on the nature of the NStim (and hence the nature of resulting *B* processes), whereas prevention relief will be uniformly present as relief from fear, independent of the NStim that is feared.

**Table 3:** Paradigms implemented in the investigation of situationally defined relief (non-occurrence of expected or cessation of actual negative stimulation or event).

Facets				
Paradigm	Prevention stopping^a^	Active passive^b^	Certain uncertain^c^	Example studies
Differential conditioning: CS^+^ predicts NStim, CS^-^ predicts absence of NStim	Prevention	Passive	Variable^d^	Bromage and Scavio (1978); Baas et al. (2002)
Imagined non-occurrence of negative event	Prevention	Passive	Certain	Idson et al. (2000)
Active avoidance of NStim or event	Prevention	Active	Variable^e^	Higgins et al. (1997); Kim et al. (2006)
Imagined successful avoidance of negative event	Prevention	Active	Certain	Idson et al. (2000)
Stimulus signals possibility to avoid or stop a NStim via instrumental behavior	Prevention/stopping	Active	Uncertain	Weiss and Schindler (1989), Weiss et al. (1996)
Presentation of stimuli signaling successful avoidance or stopping of NStim	Prevention/stopping	Active	Certain	Cándido et al. (2004); Eder and Dignath (2014)
Backward conditioning: presentation of CS^+^ after NStim (i.e., during offset of NStim)	Stopping	Passive	Certain	Walasek et al. (1995); Andreatta et al. (2013)
Measurement of dependent variables after NStim (i.e., during offset of NStim)	Stopping	Passive	Certain	Amsel and Maltzman (1950)
Active stopping of pain (e.g., pressure) when wished or at maximum tolerance level	Stopping	Active	Certain	Bresin et al. (2010)

*Aversive CS^*+*^ refers to a stimulus which predicts the occurrence of a negativeevent/stimulation*.

*^a^Cessation of experienced NStim (stopping) vs. prevention of NStim (prevention); ^b^relief independent of subject’s behavior (passive) vs. relief caused by subject’s behavior (active); ^c^level of perceived certainty of the non-occurrence or cessation of NStim; ^d^depends on the specifics of the conditioning procedure; ^e^depends on the specifics of the behavior and on the time of measurement (i.e., during avoidance or after successful avoidance)*.

**FIGURE 3:**
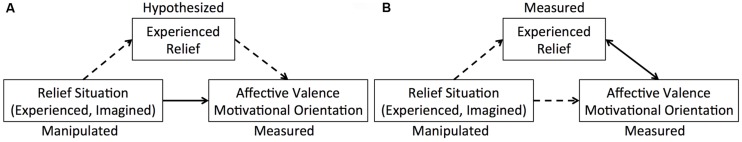
**Situation based **(A)** and experience based **(B)** research strategies.** Solid lines represent the main empirical inference, dashed lines represent hypothesized or optionally measured relations.

### INDIVIDUAL DIFFERENCES

The process assumptions of IRMO provide a map for potential precursors of individual differences in relief. Such individual differences may reside in cognitive, affective, and motivational variables. For example, factors biasing probability judgments (Gilovich et al., 2002) can be predicted to affect relief. In the NStim phase, a bias toward increased expectancies of NStim will later increase relief. In the engaged relief phase, a bias toward increased expectancies of NStim will decrease relief unless the prevention or stopping of NStim is rendered fully certain by situational or internal factors (e.g., destruction of a threatening object; full behavioral control over threat). In IRMO, relief is assumed to result from an interplay of negative, avoidance related *A* processes and positive, approach related *B* processes. Given that *B* processes are predicted to be partially determined by the intensity of *A* processes, variability in trait negative affectivity as well as avoidance motivation can be expected to positively correlate with relief. At the same time, variability in trait positive affect and approach motivation can also be expected to correlate with relief, given that they influence the arousability of *B* processes in the realm of prevented or stopped negativity.

Moreover, stronger avoidance motivation might lower an individual’s fear threshold, resulting in more frequent and intense fear, and accordingly, in more frequent and intense prevention relief due to the operation of opponent processes. Alternatively, stronger avoidance motivation might manifest itself in higher perceived probabilities of NStim. A final possibility is that stronger avoidance motivation is associated with a more efficient search for safety signals or appropriate avoidance behaviors in the NStim phase (cf. Shah and Higgins, 2001). People may also differ in their perceptual sensitivity toward specific NStim. A higher sensitivity can be expected to result in higher levels of relief.

## EVIDENCE

In what follows, we will review evidence that is informative on the validity of some predictions derived from IRMO. As we will see, however, the existing evidence rarely is based on experimental comparisons of the critical procedural features of relief that, according to our framework, will correspond with different processes and hence different motivational orientations. As such, we are almost exclusively dependent on cross-experimental comparisons. Another issue rendering existing evidence ambiguous is the lack of agreed-upon inductions of relief and measurements of motivation and affect in relief situations. For the present review, we included studies pursuing one of two research strategies. The first is a situation-based strategy that experimentally creates relief situations (see **Table 3** for an overview). Experimental paradigms to induce the offset of experienced or the prevention of expected NStim include various learning protocols, such as differential aversive conditioning, where a reinforced CS^+^ signals NStim, whereas a non-reinforced CS^-^ signals the absence of NStim. Some theorists therefore refer to this situation as conditioned relief (Gray and McNaughton, 2000, p. 55).

Besides situational inductions based on real hedonic experiences in the experimental setting (e.g., pain, noise), some studies rely on the mere imagination of relief situations (e.g., Idson et al., 2000), thereby modeling general imagination-based techniques of emotion induction (Lench et al., 2011). Often, studies using this first strategy do not include additional measures of subjective experiences, but instead investigate other correlates of relief. For example, Leknes et al. (2013) were interested in how the context in which pain relief was experienced affects the hedonic quality (positive vs. negative) of relief. To achieve this, participants experienced the offset of a heat stimulus in various contexts, and subjective ratings of hedonic pleasantness as well as various biological measures were sampled. However, no direct ratings of subjective relief were taken. Instead, the presence of relief was assumed based on the strong situational induction (see **Figure 3A**). Similar assumptions are necessary in animal studies, where the presence of relief is mainly inferred from the situational conditions (e.g., Navratilova et al., 2012). The second research strategy relies on subjective measures of relief in neutral, experimental, or imagined relief situations (e.g., Ellsworth and Smith, 1988; Leknes et al., 2008), and may then use subjective ratings as predictors of other variables of interest (see **Figure 3B**).

We included studies using various operationalizations of affective valence and approach vs. avoidance motivation ^4^. Measures of approach/avoidance motivation include the tendency to physically move toward a relief situation or away from it (see Krieglmeyer et al., 2013; Phaf et al., 2014), and the modulatory effects of relief situations on appetitive behavior (e.g., eating) or aversive behavior (e.g., fleeing) as in Pavlovian-instrumental transfer (PIT; e.g., Holmes et al., 2010). A similar variety of measures exists for stimulus valence. For example, the valence of relief situations has been assessed via self-report or its reinforcing effect on instrumental behavior.

Unfortunately, specificity for and sensitivity to valence vs. approach/avoidance motivation still remain unclear for some widely used measures. One example is eye-blink- and postauricular-startle modulation (Lang et al., 1990, 1992), which by some authors is classified as a measure of affective valence (e.g., Franklin et al., 2013b) and by other authors as a measure of motivational orientation (e.g., Lang et al., 1990; Peterson and Harmon-Jones, 2012) or the rewarding nature of a stimulus (e.g., Andreatta et al., 2013). Likewise, some brain structures [e.g., amygdala (AMY) and nucleus accumbens (NAcc)] are often interpreted as reflecting a specific affective valence (e.g., AMY and negative affect), or motivational functions (e.g., NAcc and reward processes). Yet some recent evidence casts doubt on a simple relation between activation of these structures and valence or motivation. For example, NAcc activity is often interpreted as a reward response, but there is some debate whether activation or deactivation reflects reward (e.g., Carlezon and Thomas, 2009). Furthermore, recent evidence suggests that NAcc activity generally codes motivational relevance or intensity (e.g., Jensen et al., 2003, 2007; Levita et al., 2009). Likewise, recent evidence suggests that different neuron-populations in the AMY code reward, punishment, and non-punishment (e.g., Genud-Gabai et al., 2013; Sangha et al., 2013). To deal with this issue, we will review results deriving from these measures separately in each of the following sections.

### CERTAINTY

As outlined above, IRMO suggests that different levels of certainty relate differently to the affect and motivation associated with relief. Studies that were inspired by appraisal theories give an ambiguous picture of the association between the experience of relief and certainty, and are uninformative on the question of affective and motivational underpinnings. By and large, these studies suggest that relief sometimes goes along with appraisals of high certainty (Roseman, 1984, 2013; Frijda et al., 1989; Reisenzein and Spielhofer, 1994), sometimes with appraisals of low certainty (Roseman et al., 1990; Tong, 2015), or is unrelated to certainty (Ellsworth and Smith, 1988; Roseman, 1991). These diverging results may be due to the fact that often rather broad measures of certainty were taken, and that the affective vs. motivational facets of relief were not separated in the measures of subjective relief.

#### Affective valence and startle modulation

In an experimental study drawing on aversive Pavlovian conditioning, Andreatta et al. (2013) examined the subjectively rated affective valence of stimuli associated with threat (forward CS^+^) and stimuli associated with the situation-caused stopping of negativity (backward CS^+^) by using subjective ratings of valence as well as eye-blink startle modulation as dependent variables. Importantly, the stopping relief stimuli were either perfectly predicted by forward CS^+^, or occurred after painful stimulation that was not signaled by preceding stimuli. While the stopping relief could be predicted even before the onset of pain in the first condition, no such anticipation of relief was possible in the second condition. Results indicate that the predictable stopping relief stimulus was subjectively positive. Also, this stimulus decreased startle reactivity below baseline, whereas the unpredictable stopping relief stimulus was subjectively negative and increased startle reactivity. A study by Leknes et al. (2011) provides further evidence on the role of certainty for the affective valence of relief based on personality variables. More specifically, they observed a positive correlation between trait pessimism (a trait proxy for certainty of being punished) and (a) the anticipatory fear of being punished, and (b) the subjectively reported pleasantness of relief after passive pain relief. These observations support the theoretical notion that certainty of being punished increases anticipatory negative affect, and that the intensity of anticipatory negative affect positively influences the intensity of relief.

### PREVENTION vs. STOPPING

For the engaged relief phase, the following expectations can be derived from IRMO: (A) stopping compared to prevention relief goes along with stronger approach relative to avoidance motivation; (B) stopping compared to prevention relief goes along with more positive relative to negative affect. Typical experimental inductions of prevention relief are presenting CS^-^ in aversive Pavlovian conditioning, or training participants in instrumental avoidance behavior. Typical experimental inductions of stopping relief involve applying a pain stimulus and then removing it, either by or without participants’ behavioral intervention. Dependent variables are either measured immediately after removal of the NStim or in response to stimuli that were systematically paired with the experience of stopping relief. Unfortunately, we did not find any empirical studies informative on potential differences in certainty appraisals as a function of prevention vs. stopping relief. We therefore have to focus on measures of affective valence, motivational orientation, and measures probably tapping into both valence and motivational orientation.

#### Affective valence

A relatively large literature on prevention relief concerns the valence of CS^-^_aversive_ (Rescorla, 1969; Gray, 1971; Savastano et al., 1999). Many studies of this kind suggest that CS^-^_aversive_ are evaluated more favorably than CS^+^_aversive_ (e.g., Baas et al., 2002). However, studies comparing CS^-^_aversive_ to a neutral control condition rather indicate that CS^-^_aversive_ just became less aversive, but not more positive than control (Lipp et al., 2003; Mallan and Lipp, 2007). This is also supported by the results of an animal study by Fernando et al. (2013), who found safety stimuli to be just as rewarding as control stimuli, while at the same time being less rewarding than appetitive stimuli. Human studies using hypothetical relief from monetary punishment point into a similar direction (Idson et al., 2000). A study by Andreatta et al. (2012), however, suggests that CS^-^_aversive_ were positive in the sense that they were rated above the scale midpoint, but at a similar level as control stimuli that were never presented during learning. As such, the above-midpoint rating may not reflect a learning-based increase in positivity in CS^-^_aversive_, but rather context-effects during test. With stopping relief, there is more evidence in favor of positive valence. For example, Zanna et al. (1970) observed that words associated with the stopping of electro shocks were evaluated more positively than baseline. Leknes et al. (2013) observed that a reduction in pain was evaluated as positive above a well-defined neutral anchor of visual analogue scales. A similar result was observed for stimuli associated with the offset of pain in one study (Andreatta et al., 2013). Yet, there is also contradictory evidence. Two studies yielded more negative evaluations compared to a pre-conditioning baseline for stimuli associated with stopping-relief (Andreatta et al., 2010, 2012), and another study observed a decrease in positive as well as negative self-reported affect after pain offset (Bresin and Gordon, 2013).

#### Motivational orientation

One method to study how prevention relief affects approach/avoidance motivation draws on PIT (Rescorla and Solomon, 1967). For example, Rescorla and Lolordo (1965) trained dogs to differentiate between danger- and safety-stimuli in the context of receiving electric shocks. Also, the dogs trained behavior instrumental to avoid shock. In a test phase, danger and safety stimuli were presented during the instrumental avoidance behavior. As a result, danger stimuli increased whereas safety stimuli decreased instrumental avoidance behavior. Apparently, the safety stimuli had acquired the potency to suppress fear and/or avoidance motivation. Many other studies support the notion that safety signals reduce instrumental avoidance behavior (e.g., Arcediano et al., 1996), and may increase appetitive instrumental behavior (Ray and Stein, 1959). Other evidence, however, suggests that this latter effect is presumably weak and highly moderated (e.g., Hoffman and Fleshler, 1964; Hammond, 1966). Evidence drawing on different methods to assess the motivational nature of prevention relief adds to this ambiguity. While some studies bolster the notion that safety stimuli boost appetitive motivation (Bromage and Scavio, 1978), other studies again suggest that this is highly moderated (DeVito and Fowler, 1994). Additionally, there is evidence that safety stimuli induce approach motivation in the sense that animals develop a preference for the place of their occurrence (Rogan et al., 2005) and are faster to run toward prevention relief signals (Haraway et al., 1984).

Observations regarding the motivational properties of stopping relief are often based on a backward conditioning paradigm. In such studies, stimuli are presented together with the offset of NStim. Experimentally, such stopping relief stimuli gain the power to inhibit avoidance behavior (Moscovitch and LoLordo, 1968; Grelle and James, 1981; Cole and Miller, 1999), and are also approached by animals (Tanimoto et al., 2004; Yarali et al., 2008). Stopping relief might also facilitate appetitive behavior. Drawing on animal research subjects, Amsel and Maltzman (1950) observed increased drinking behavior after stopping relief compared to baseline. Similar observations were made in other studies (Davis et al., 1976; Walasek et al., 1995), but another study found stopping relief stimuli to be no more appetitive than neutral control stimuli (Krank, 1985).

#### Startle modulation

Mirroring the observations with subjective valence measures, numerous studies indicate that stimuli associated with prevention relief are less aversive than threat stimuli, but still more aversive than baseline (e.g., Hamm et al., 1993; Falls and Davis, 1995; Lipp et al., 2003; Josselyn et al., 2005; Jovanovic et al., 2006; Mallan and Lipp, 2007; Weike et al., 2008). For stopping relief, available evidence provides a different picture. For example, Franklin et al. (2013a) observed that post-auricular startle was enhanced and eye-blink startle was reduced after pain off-set compared to baseline. Given that post-auricular startle may be interpreted as an indicator of approach motivation or positive valence (Benning et al., 2004; Gable and Harmon-Jones, 2009), this suggests that stopping relief is more than a mere reduction in aversive motivation or negative valence, but instead shifts the affective-motivational tone toward positive or approach. Similar findings were obtained in a number of other studies (Leknes et al., 2008; Andreatta et al., 2010, 2012, 2013; Franklin et al., 2010, 2013b).

#### fMRI studies

Leknes et al. (2011) investigated brain-activation patterns associated with prevention relief and reward. Functional magnetic resonance imaging (fMRI) analyses demonstrated some brain regions (ventromedial prefrontal cortex, rostral anterior cingulate) to jointly respond to prevention relief and imagined reward, whereas other regions were either specific to relief (e.g., right anterior insula, NAcc) or to imagined reward (e.g., posterior cingulate). This suggests that prevention relief shares some processes with reward but also has some distinct features. A similar conclusion can be drawn from a study by Genud-Gabai et al. (2013), who observed that neuron populations in the AMY not only respond to fear stimuli, but likewise to safety stimuli in a prevention setting. Sangha et al. (2013) compared neural responses to stimuli signaling shock, safety, or reward (sucrose). They observed that about 18% of the recorded neurons responded to both threat and prevention relief signals. There were two other neuron populations in the basal AMY, one responding selectively to prevention relief signals, another one selectively firing to prevention relief and to reward signals. This pattern may be interpreted as further evidence for the ambivalent nature of the reactions triggered by prevention relief signals, overlapping with reactions to threat stimuli (aversive component), and reward stimuli (appetitive component), and including a component idiosyncratic to prevention relief. A recent study drawing on experimentally inflicted pain and pain relief in animals suggests that stopping relief corresponds with dopamine release in the NAcc (Navratilova et al., 2012), which is often interpreted as implying a rewarding nature. Corroborating this finding, Andreatta et al. (2012) observed increased activity in the ventral striatum in human participants in response to CS associated with pain offset (i.e., stopping relief) compared to control stimuli.

### ACTIVE vs. PASSIVE

The following expectations can be derived from IRMO for the engaged relief phase: (A) Active relief goes along with high avoidance motivation, passive relief results in reduced avoidance motivation and possibly increased approach motivation; (B) Outcomes of active relief that signal a safety period induce processes similar to passive relief; (C) Active and passive relief are of positive affective valence through opponent processes, with active relief possibly being more positive due to enhanced attention and controllability appraisals; (D) Actively generated CS^-^_aversive_ inhibit negative affect and avoidance motivation more strongly than passively learned CS^-^_aversive_. Based on OPT, this will result in more positive affect and approach motivation. Affective and motivational effects of passive relief may be measured during the experience of the offset of NStim, or in response to stimuli associated with prevented or eliminated NStim. Active relief, on the other hand, can be studied in operant settings where participants learn to avoid negativity through engaging in specific behaviors. A simpler variant excludes learning processes, such as in active pain relief where participants can simply stop the application of pain, for example by voluntarily removing their hand from ice water. As with prevention vs. stopping, often cross-study comparisons are necessary to evaluate possible differences between the two types of relief.

#### Affective valence

Numerous studies show that passive prevention relief is less negative than threat, albeit still negative when compared to baseline (e.g., Mallan and Lipp, 2007). Passive stopping relief, however, sometimes turned out to be more positive than a neutral baseline (Andreatta et al., 2013) although the evidence is mixed (Andreatta et al., 2010; Bresin and Gordon, 2013). Evidence suggests S^D^_relief_ as well as self-produced safety signals to be of positive valence, or at least to decrease negative affect. Murray and Strandberg (1965) observed that self-produced safety signals had reinforcing properties in rats, and Kinsman and Bixenstine (1968) demonstrated such reinforcing effects to be stronger for self-produced safety signals than for S^D^_relief_. This difference is in line with the predictions of IRMO. A study by Eder and Dignath (2014), which compared stimuli associated with passive and active (prevention) relief, observed that active and passive relief result in different levels of positivity. Colors which signaled the successful outcome of active avoidance behavior were rated as positive on both an explicit and an implicit measure of valence, whereas colors which signaled passive relief were rated as positive on an explicit, but not an implicit measure of valence. Accordingly, this study indicates that the outcome of active prevention relief might be more consistently positive than CS^-^_aversive_. Similar results were obtained by Niznikiewicz and Delgado (2011), who observed greater positivity along with higher emotional intensity for active relief compared to passive relief on an explicit self-report measure.

#### Motivational orientation

The integrative relief model assumes that, during the engaged relief phase, CS^-^_aversive_ will inhibit avoidance motivation, whereas S^D^_relief_ will not. The inhibiting effect of passive relief stimuli is attested by the inhibiting effect of passive and self-generated CS^-^_aversive_ on instrumental avoidance behavior (e.g., Rescorla and Lolordo, 1965; Berger and Starzec, 1988; Arcediano et al., 1996). However, IRMO suggests actively produced safety signals to have stronger inhibiting effects than passive relief stimuli. Relevant evidence for this prediction comes from a study by Cándido et al. (2004), who compared the effects of stimuli signaling successful avoidance and pf passive relief stimuli on the intensity of an independent, secondary fear response. Results suggest that actively produced safety signals suppressed fear more intensely than passive relief stimuli.

The integrative relief model also predicts active relief to go along with high avoidance motivation, whereas passive relief is predicted to reduce avoidance motivation. Supporting evidence for the first part of the prediction comes from studies by Weiss and Schindler (1989) and Weiss et al. (1996). In one study (Weiss et al., 1996), rats in one condition learned that a first discriminative stimulus (e.g., a clicker) signaled that they could gain food by pressing a bar, whereas a second discriminative stimulus (e.g., a tone) signaled that they could postpone an aversive shock by pressing on the bar. Importantly, in a test phase, both discriminative stimuli were presented simultaneously. Results indicate that the compound of an active relief and an active joy (i.e., reward attainment) stimulus resulted in decreased bar pressing compared to a compound of two joy or two relief stimuli. Thus, while the motivational power of two stimuli with identical incentives (joy/joy; relief/relief) added up, this was not the case for mixed joy/relief stimuli. This suggests that the possibility to avoid shock by bar pressing and the possibility of gaining a reward through bar pressing were associated with the activity of different motive systems, providing indirect support for a lack of inhibition of avoidance motivation through active relief. Similar results were reported by Weiss and Schindler (1989).

Frijda et al. (1989) observed that participants who were asked to recall a situation in which they had experienced relief rated the situation as high in self-agency, an appraisal dimension we associate with active relief, and as high on the motivation to approach. While this may imply that active relief will activate an approach motivational orientation, it should be emphasized that the methodology of the study does not allow for a certain statement about the phase in the relief process that participants’ appraisal of approach motivation refers to, nor about whether it refers to a safety period established by active avoidance or not.

#### Startle modulation

Regarding the modulation of eye-blink startle, evidence suggests passive prevention relief to still have aversive qualities, albeit being less aversive than NStim (e.g., Mallan and Lipp, 2007), while passive stopping relief was positively associated with approach or positive valence. A study which investigated active stopping relief also observed decreased eye-blink startle reactivity relative to baseline, indicating a strong reduction in negative affect or avoidance motivation by successful active stopping relief (Franklin et al., 2010). Although IRMO does not predict a reduction in avoidance motivation through active relief, these results are still compatible with IRMO under the assumption that the period after pain cessation was experienced as a safety period by participants. These self-produced safety periods or safety signals are predicted to reduce negative affect and avoidance motivation.

#### fMRI studies

A study by Levita et al. (2012) examined activity in the AMY and the NAcc in response to situations in which participants could actively avoid NStim or NStim did not occur when participants remained passive. Results indicate that active avoidance caused an increase in (primarily right) NAcc activity, whereas passive avoidance caused a decrease. Moreover, state anxiety predicted NAcc activation and deactivation. A similar pattern was observed for (primarily right) AMY activation, which was increased in active but decreased in passive avoidance (for a comparable finding, see Delgado et al., 2009). Similarly, Schlund et al. (2010) observed AMY activation to be increased during active avoidance compared to a neutral control, and the intensity of avoidance behavior to be positively correlated with AMY activity. Relatedly, Kohls et al. (2013) observed increased NAcc activation when participants prepared to avoid negative outcomes compared to a control condition and compared to the situation when the negative outcome was finally avoided. Typically, NAcc activity is interpreted as a reward response, but recent evidence is more compatible with the notion that NAcc activity generally codes motivational relevance or intensity (e.g., Jensen et al., 2003, 2007; Levita et al., 2009). This also better fits the dissociation between active and passive avoidance observed in Levita et al. (2012). From this perspective, the most conservative interpretation of these studies would imply stronger motivation in the case of active vs. passive relief.

Although it does not bear on the comparison between active and passive relief, a study by Kim et al. (2006) is informative regarding a distinction in IRMO between two different steps in the process of active relief during the engaged relief phase, namely appraising that an avoidance behavior can be performed in response to the expectancy of NStim, and the appraisal that the executed avoidance behavior reduced the expectancy of NStim. According to IRMO, the latter should be associated with more positive affect than the former, since the expectancy of NStim is further reduced as a result of successful avoidance. Kim et al. (2006) used a choice task in which participants could increase their chances of attaining a reward (a monetary gain) or avoiding a negative outcome (a monetary loss) by making the correct choice between two stimuli. They found that successfully avoiding a negative outcome in the choice task increased neural activity in the medial orbitofrontal cortex, a region associated with encoding the reward value of stimuli (Kim et al., 2006), just like actively attaining a reward, indicating that successfully avoiding a negative outcome, just like successfully attaining a positive outcome, is rewarding. However, a different picture emerged for brain activity at the time of choice, i.e., when avoiding NStim (or attaining a reward) was possible, but no feedback about the success of the avoidance (or reward attainment) was yet received. Here, activity in regions found to correlate with the expectation of a future rewarding outcome (the medial and lateral orbitofrontal cortex) decreased over time for trials in which a negative outcome could be avoided, while activity in regions associated with expectations of future aversive outcomes (the right dorsolateral prefrontal cortex and the anterior cingulate cortex) increased over time. The reverse pattern was found for trials in which a positive outcome could be achieved. These results indicate negative affectivity during an avoidance process prior to feedback about the outcome of avoidance behavior, and positive affectivity after the receipt of feedback indicating successful avoidance.

### INDIVIDUAL DIFFERENCES

Some studies provide evidence on the relation between relief and individual difference variables. Based on OPT mechanisms, IRMO suggests an association of relief with both trait avoidance and trait approach. In line with this assumption, one study observed the experience of relief to be associated with an individual’s chronic level of avoidance motivation. Specifically, Higgins et al. (1997) observed that avoidance motivation increased the impact of NStim on the frequency and intensity of quiescence-related emotional experiences (which include relief). Similar results were obtained for chronic avoidance motivation and failure on a task (Idson et al., 2000) or outgroup members (Shah et al., 2004) as NStim. Additional studies investigating chronic as well as situationally induced avoidance motivation observed similar results (e.g., Higgins and Tykocinski, 1992; Shah, 2003; Falomir-Pichastor et al., 2008; Yi and Baumgartner, 2008; Adams et al., 2011; Falomir-Pichastor et al., 2011). However, there are also a few studies that did not show this pattern or even a contradictory mapping of emotional tone and motivational orientation (Faddegon et al., 2008; Yi and Baumgartner, 2009; Winterheld and Simpson, 2011; McKay-Nesbitt et al., 2013).

Other studies also provide evidence on the role of approach related dispositions. In two studies, Carver (2009) measured BAS and BIS strength and assessed their impact on felt relief by using a scenario technique. Results revealed that the intensity of relief was positively correlated with both the strength of the BIS, as well as with one subscale of the BAS, namely reward responsiveness, but that the association between the BIS and relief was stronger than the association between reward responsiveness and relief, thereby supporting the dual nature of relief as predicted by the OPT assumptions of IRMO. Another study drawing on a conditioned inhibition paradigm even suggests prevention relief to be solely related to BAS reward responsiveness and no other components of the BIS/BAS questionnaire (Migo et al., 2006). However, some studies have failed to find associations between the experience of relief or other quiescence-related emotions and chronic approach or avoidance motivation (Leone et al., 2005; Yen et al., 2011). Leknes et al. (2011) measured individual differences in the subjective pleasantness of appetitive reward scenarios. There was a substantial positive correlation between the pleasantness of actual pain relief and the pleasantness of appetitive reward, further corroborating a link to approach related dispositions. Leknes et al.’s (2011) study is also informative as to the role of trait variables related to probability estimates and to the fear threshold. As would be expected, trait pessimism was positively correlated with relief and with acute dread. Interestingly, pessimism and dread did not correlate with appetitive reward.

## IMPLICATIONS FOR THEORY AND RESEARCH

We started out by describing how theories of relief converge and differ regarding the affective valence, and motivational orientation associated with relief (cf. Carver, 2009). Some theories suggest relief to be part of the BAS (e.g., Gray, 1987; Gray and McNaughton, 2000), whereas other theories conceptualize relief as a positive emotion of avoidance processes (e.g., Carver and Scheier, 1990, 2002; Carver, 2001). We proposed an integrative model, IRMO, that combines process assumptions and conceptual distinctions from a number of existing theories. Based on IRMO, we derived a number of predictions regarding the moderating nature of features related to the relief situation (certainty, active vs. passive, prevention vs. stopping) on the affective and motivational nature of relief. Unfortunately, systematic empirical research on moderators is rather scarce. Nevertheless, a preliminary evaluation of the validity of some of IRMO’s predictions is possible, mostly based on cross-study comparisons though.

### PREVENTION vs. STOPPING

The integrative relief model predicts stopping relief to trigger more positive affect and approach motivation than prevention relief. The rationale for this prediction was that stopping relief has a strong experiential component of the factual offset of NStim, whereas prevention relief does not. This may also correspond with the fact that stopping always entails a proof of being factual, whereas prevention relief is anticipatory and may still come with some degree of uncertainty. In line with this reasoning, reviewed evidence on the valence of CS^-^_aversive_ as a proxy for prevention relief suggests that while such stimuli might be rated as being more positive than CS^+^_aversive_ (Baas et al., 2002), CS^-^_aversive_ seem to show little positivity and approach motivation compared to neutral control conditions, and fMRI evidence points to the possibility that CS^-^_aversive_ activate representations of punishment and reward at the same time. With stopping relief, however, the reviewed evidence implies different regularities. Drawing on startle modulation as a dependent variable, evidence suggests that eye-blink startle reactivity decreases below baseline during stopping relief or in the presence of stimuli associated with stopping relief. Moreover, post-auricular startle – a marker of positive affect or approach motivation – as well as activation in reward associated brain regions increased during stopping relief. This evidence, although relying on cross-experimental comparisons, is supportive of the predictions derived from IRMO.

Evidence drawing on the modulation of instrumental behavior is less clear, however. There clearly is evidence that passive prevention relief decreases instrumental avoidance behavior, and, perhaps to a weaker degree, increases instrumental approach behavior. There is also clear evidence that stopping relief inhibits avoidance behavior and facilitates approach behavior. Both main effects are compatible with IRMO, as the mechanisms predict both prevention and stopping relief to reduce avoidance motivation and hence, by means of OPT, increase approach motivation. The more distinctive prediction (i.e., stronger shift toward approach for stopping), however, presupposes a within-experiment manipulation of prevention vs. stopping relief, which, according to our search, is still missing.

### ACTIVE vs. PASSIVE

The integrative relief model predicts that during the engaged relief phase, both active and passive relief will be associated with an increase in positive affect, with a potentially stronger effect for active relief. Active and passive forms of relief are expected to differ, however, with regard to their association with approach and avoidance motivation. Whereas passive relief is predicted to go hand in hand with a shift toward approach motivation, this is not expected to be the case for active relief. Rather, active relief is assumed to involve the activation of avoidance behavior, and a strengthening of this behavior if avoidance successfully reduces the expectancy of NStim. The exception to these predictions, however, are signaled safety periods produced as a result of active avoidance, which are assumed to exert the same effects as passive relief – i.e., increase in positive affect and approach motivation – albeit to a stronger degree due to processes such as illusions of control which favor actively produced over passively endured outcomes. The results reviewed in this article support these predictions for the most part. While there is evidence for the positivity, or at least decreased negativity, of both passive and active relief, successful active relief was indeed found to be more consistently positive than passive relief (Eder and Dignath, 2014). Cross-experimental comparisons appear to further support this point, as stimuli associated with successful active relief were found to reinforce the acquisition of a novel response (Murray and Strandberg, 1965; Kinsman and Bixenstine, 1968), whereas stimuli associated with passive relief were not (Fernando et al., 2013).

Regarding the effect of active and passive relief on approach and avoidance motivation, passive relief was indeed found to be associated with a decrease in avoidance motivation (Rescorla and Lolordo, 1965), whereas ongoing active relief was not (e.g., Weiss et al., 1996). Moreover, successful avoidance strengthens the avoidance behavior that led to the avoidance or escape from NStim (Dinsmoor, 2001). During the presence of stimuli associated with a self-produced safety period, however, avoidance motivation was reduced to a stronger degree than during the presence of stimuli associated with passive relief (Cándido et al., 2004), as predicted by IRMO. While it comes with some ambiguity, one plausible interpretation of fMRI studies (Delgado et al., 2009; Levita et al., 2012; Kohls et al., 2013) is that active relief goes along with greater avoidance motivation than passive relief.

### IMPLICATIONS FOR VALENCE- vs. GOAL-THEORIES

The observed patterns of results are relevant for evaluating the viability of valence theories (e.g., Gray, 1987; Gray and McNaughton, 2000) and goal theories (e.g., Carver and Scheier, 1990, 2002; Carver, 2001) of emotion for explaining the affective and motivational underpinnings of relief. These classes of theories uniformly associate relief with positive affect, but valence theories associate relief with approach motivation, whereas goal theories associate relief with avoidance motivation (Carver, 2009). As our review demonstrated, neither prediction fully matched available evidence. Some studies indicate that relief stimuli are of negative valence and avoidance motivation albeit less so than fear signals (e.g., Hamm et al., 1993; Falls and Davis, 1995; Lipp et al., 2003; Josselyn et al., 2005; Jovanovic et al., 2006; Mallan and Lipp, 2007; Weike et al., 2008), whereas other studies suggest that relief and associated stimuli are more positive than baseline or control stimuli and associated with approach motivation (e.g., Dinsmoor and Sears, 1973; Andreatta et al., 2010, 2013; Franklin et al., 2013a; Eder and Dignath, 2014). fMRI based studies demonstrate on the one hand some overlap of relief and reward (Kim et al., 2006; Leknes et al., 2011; Genud-Gabai et al., 2013; Sangha et al., 2013). At the same time, relief goes along with brain-activation that is specific for relief and independent from reward (Leknes et al., 2011; Genud-Gabai et al., 2013; Sangha et al., 2013), and some neuron populations respond to both threat and relief at the same time (Kim et al., 2006; Genud-Gabai et al., 2013; Sangha et al., 2013). Studies drawing on personality measures corroborate a heterogeneous nature of relief when it comes to motivational orientations, suggesting that it contains both approach and avoidance components (Migo et al., 2006; Carver, 2009). Clearly, relief is neither only positive, nor only negative. And it does not unambiguously match with approach or avoidance. This suggests that goal- and valence-theories might profit from extensions so that they can accommodate the more differentiated empirical patterns observed so far and – optimally – generate novel predictions. IRMO can be interpreted as such an attempt.

### RELATION TO FEAR, FRUSTRATION, AND HOPE

Relief is not the only emotion showing complex relationships with affect and motivational orientation. A similar picture emerges for emotions resulting from frustration situations such as anger or sadness. Frustration situations are situations in which an expected or experienced positive stimulation is reduced or absent (cf. Dollard et al., 1939; Berkowitz, 1989; Papini and Dudley, 1997). As such, frustration situations are the mirror image of relief situations. Clearly, emotions resulting from frustration situations such as anger and sadness have negative valence. Whether frustration situations are associated with an approach or avoidance motivation, however, is less clear. On the one hand, empirical evidence indicates a relation between anger and approach motivation (Carver and Harmon-Jones, 2009). On the other hand, frustration situations have been shown to trigger avoidance behavior in animal studies (Papini and Dudley, 1997). As with relief, various moderators may determine which motivational orientation is triggered by frustration situations. In particular, appraised control or coping potential determines the nature of emotional responses to frustration situations (Wortman and Brehm, 1975; Roseman, 2001; Smith and Kirby, 2001) and may thus also determine whether an approach or avoidance motivation is activated. For instance, research investigating hemispherical lateralization as an indicator of motivational orientation has shown that coping potential moderates the extent to which anger-inducing situations elicit an approach motivation (Harmon-Jones et al., 2003). Furthermore, appraisals of agency influence the extent to which anger arises (Roseman, 1991) and may thus influence the extent to which approach motivation is triggered. However, empirical evidence on the moderating influence of appraisals on motivational orientation is scarce. If one were to apply IRMO to frustration, one could conceptualize the appraisal of high coping potential as a situation where the probability of attaining a positive outcome is higher when performing a behavior than when not performing the behavior, in analogy to active relief.

While frustration, anger and sadness are mirror images of relief, fear is one potential precursor of relief. IRMO conceptualizes prevention relief as a reduction in fear, more specifically a reduction in the probability of experiencing NStim conditional on the availability of safety signals or avoidance responses. Prevention relief therefore goes hand in hand with a reduction in fear. Note, however, that IRMO suggests that the avoidance motivation triggered by fear will decrease through passive relief. But IRMO also suggests the avoidance motivation to continue even after reduction in fear for active relief during the engaged relief phase. As already hypothesized in several theories (e.g., Ortony et al., 1988; Reisenzein, 2009), we predict that the intensity of relief will be related to the intensity of antecedent fear. IRMO offers several reasons why this might be the case. First of all, more intense fear will result in a stronger *A* process, which will result in a stronger *B* process, and consequently larger residual activity of the *B* process in the disengaged relief phase. Moreover, more intense fear, conceptualized as a higher perceived probability of NStim, will make relative relief more likely. This is expected to be the case because even if safety signals or the availability of avoidance responses are associated with a probability of NStim that is still high in absolute terms, the difference between this probability and the probability of NStim in the absence of safety signals or avoidance responses might be rather large in the case of intense fear. Even in the case of stopping relief, which is driven by actual instead of by expected NStim, the possibility that pain might return could trigger anxiety, which will motivate the search for relief cues or adequate avoidance responses during the engaged relief phase. IRMO predicts a complete absence of fear and anxiety only in the disengaged relief phase.

**Table 4:** Exemplary measures of valence and of approach and avoidance motivation implemented as dependent variables.

Measured construct	Dependent variable	Example studies
Valence	Self-report of valence	Roseman (1996); Baas et al. (2002)
Valence	Affective priming task	Eder and Dignath (2014)
Valence or motivational orientation	Eye-blink startle modulation	Josselyn et al. (2005); Andreatta et al. (2013)
Valence or motivational orientation	Post-auricular reactivity modulation	Franklin et al. (2013a,b)
Valence, motivational orientation, motivational intensity, or relevance	fMRI: ventral striatum/nucleus accumbens; fMRI: amygdala	Leknes et al. (2011); Genud-Gabai et al. (2013)
Valence	Reinforcement of instrumental behavior through stimulus associated with relief	Fernando et al. (2013)
Motivational orientation (behavior)	Reinforcement of avoidance behavior (relief as consequence of behavior)	Dinsmoor and Sears (1973)
Motivational orientation	Self-report of approach action tendency	Frijda et al. (1989)
Motivational orientation	Self-report of motive to avoid punishment	Roseman et al. (1990), Roseman (1996)
Motivational orientation	Preference for place of occurrence	Rogan et al. (2005)
Motivational orientation	Latency and likelihood of moving toward safety stimulus	Haraway et al. (1984)
Motivational orientation	Decrease in fear response (e.g., freezing)	Cook et al. (1987)
Motivational orientation	Decrease in the inhibiting effect of aversive CS^+^ on appetitive behavior (e.g., drinking)	Cándido et al. (2004)
Motivational orientation	Pavlovian-instrumental transfer (PIT): increase/decrease of instrumental avoidance behavior by relief stimuli	Rescorla and Lolordo (1965), Arcediano et al. (1996)
Motivational orientation	PIT: increase/decrease of instrumental appetitive behavior by relief stimuli	Ray and Stein (1959); Davis et al. (1976), Walasek et al. (1995)
Motivational orientation	Rate of performance of instrumental behavior when stimuli which signal both the possibility to gain a reward, and to avoid a NStim through the same behavior are presented	Weiss et al. (1996)
Motivational orientation	Counter-conditioning: rate of relearning of an aversive CS^-^ as an appetitive CS^+^	Krank (1985); DeVito and Fowler (1994)

Finally, hope is an emotion that might be considered in relation to relief. Based on Roseman (1984, 2013) one might consider that the degree of certainty of non-punishment corresponds to emotions ranging from fear (very uncertain non-punishment/somewhat uncertain punishment) over hope (intermediate certainty of non-punishment/punishment) to relief (certain non-punishment/no chance of punishment). From Roseman’s perspective, then, not all facets of relief as described in IRMO would actually be labeled relief. More specifically, this label would only apply to the disengaged relief phase, where conditional and unconditional threat of NStim is low. On one hand, this may be seen as an issue of labeling. One might simply decide to label the inner responses during engaged relief as hope. At the same time, this perspective would be incompatible with theory and research suggesting the existence of relative relief (Leknes et al., 2013), and the feedback-function of relief during active avoidance (Carver and Scheier, 1990).

### OPEN QUESTIONS AND AVENUES FOR FUTURE RESEARCH

#### Systematic research on certainty

The integrative relief model suggests that various certainty appraisals play an important role for the occurrence of relief. Coarsely, the certainty of NStim in the present context, as well as the conditional probability of NStim when safety cues or avoidance behavior are present, determine the three phases of relief. More specifically, IRMO predicts very specific relations between levels and changes of certainty appraisals, affect, and motivational orientation. To our knowledge, few studies have systematically manipulated or measured certainty, and we are not aware of studies that measure or manipulate all types of certainty appraisals that IRMO deems relevant for relief. At the same time, experimental manipulations of all underlying probabilities seem easily achievable and highly desirable at the same time.

#### Systematic research on types of relief

Although the present review provided some evidence for the importance of active vs. passive, and stopping vs. preventing relief, most of the conclusions were drawn from cross-study comparisons. While such comparisons are informative to some degree, they still suffer from serious threats to validity because of confounding factors. For example, stopping vs. prevention is often confounded with certainty, since it is usually quite apparent that NStim has ended, whereas NStim that has not occurred might still occur, rendering stopping relief more certain than prevention relief in a lot of cases. A potential way to solve this problem is to implement stimuli which signal that a NStim will end soon as stopping relief stimuli. This could be accomplished in a within subjects design in which subjects are presented with a NStim of a certain length in every trial, unless the trial is preceded by a safety signal (i.e., the prevention relief stimulus). Moreover, on some trials the NStim will end earlier than usual, namely a short time after the presentation of another stimulus (i.e., the stopping relief stimulus). After participants have learned the meaning of these two stimuli, their valences and effects on approach and avoidance motivation can be measured by using the stimuli as target stimuli in appropriate measures (e.g., an affective priming task, a Manikin task).

Moreover, studies on the affective and motivational consequences of relief do not always include (a) baseline or control conditions, and (b) independent measures for positive affect/approach motivation and negative affect/avoidance motivation. If these measurements are missing, it is hard to evaluate whether relief goes along with decreased negativity/avoidance motivation or increased positivity/approach motivation. For sure, some models of affect and motivation assume strict negative correlations between positive/approach on the one hand and negative/avoidance on the other hand. It would still be informative to have the opportunity to test the strength of this assumption in all experiments.

#### Diverse negativity = diverse relief?

The core of all definitions of relief is that something negative is prevented, stopped, or reduced. But negativity can come in many forms, which may result in differences in relief that follows these different sources of negativity. More specifically, based on the OPT components of IRMO, the nature of the negative *A* process determines the nature of the positive *B* process, and the interplay of the two partially determines the character of a relief episode. What are potential differences in NStim? First, evidence and theory suggests that negative affect may result from approach processes, which occurs when goal-pursuit is blocked briefly (resulting in frustration and anger, e.g., Carver and Harmon-Jones, 2009) or prolonged (resulting in sadness or depression, e.g., Higgins, 1987; Carver, 2004; Roseman, 2013). Consequently, relief, as defined here, may include the prevention or stopping of frustration. As a consequence, the underlying *A* process would be negative and of approach motivation, whereas the resulting *B* process would be positive and of avoidance motivation. Experimentally inducing relief from frustration or anger seems possible, and testing its motivational nature would help further evaluating the viability of OPT assumptions in the realm of relief. Second, there are many specific and qualitatively different NStims, such as heat, bad smell, cold, or social rejection etc., each potentially associated with diverging *A* and *B* processes. For example, Leknes et al. (2008, p. 800) theorize “A putative neurobiological mechanism for the opponent process of pain is the endogenous opioid system”. Would opioid release be the appropriate *B* process for hunger? Whereas pain may indeed trigger opioid reactions, hunger would go hand in hand with glycogenolysis or gluconeogenesis. From this perspective, the *B* processes associated with pain and hunger might partially differ, and hence different phenomenologies of relief from pain vs. relief from hunger might result. From this perspective, systematically studying differences of relief from different NStims would be a worthwhile endeavor.

#### Other measures of motivation

As documented in our review, studies on relief have drawn on an impressive number of research methods to assess affective valence and motivational orientation (see **Table 4**). However, one approach to measuring motivational orientations is surprisingly missing: cortical asymmetries as assessed by EEG recordings. Such asymmetries – both assessed in resting state as well as in response to emotion-relevant stimuli – proved to be a helpful piece of the puzzle of the motivational orientation underlying anger and sadness (Carver and Harmon-Jones, 2009). Assessments of cortical asymmetries come with the advantage of high temporal resolution and indirect measurement. Based on the experiences of research on sadness and anger, applying such measures to relief would be highly desirable.

#### Trajectory of avoidance goals

An interesting avenue for future research concerns the disengaged relief phase. On one hand, some theories of relief (e.g., Pekrun et al., 2002; Roseman, 2013) and general motivation (Förster et al., 2007) suggest that experiencing relief goes hand in hand with a deactivation of avoidance motivation or avoidance goals, and this view is also compatible with Carver’s (2009) notion of motivational reorienting after a threat is eliminated. On the other hand, recent evidence suggests that goals may remain accessible in memory after goal fulfillment until they are replaced with alternative goals (Walser et al., 2012, 2014). If the latter perspective would also apply to relief, this would suggest that the disengaged relief phase is characterized by accessible avoidance goals that are only weakly shielded against competing goals. This leads to the interesting prediction of disengaged relief resulting in an increased propensity to re-engage in earlier avoidance goals as long as they have not been replaced by other goals primed by the organism or the environment.

## SUMMARY

The present paper reviewed existing theory and evidence on the affective and motivational underpinnings of relief. The evidence suggests that relief is a heterogeneous phenomenon in that it can come with positive affect, negative affect, and ambivalent affect. Moreover, evidence suggests that relief may go along with dominant approach and dominant avoidance motivation. As such, the evidence is by and large incompatible with two broad classes of emotional theories that characterize relief as of positive valence, with valence theories linking relief uniformly with approach, and goal theories linking relief uniformly with avoidance. We also presented an IRMO that aims at integrating existing process assumptions regarding relief. It was designed to cover a large number of known effects regarding relief. With respect to affect and motivation, IRMO pointed at variants of relief, that are characterized by active vs. passive avoidance as well as stopping vs. preventing NStim. IRMO suggests that these variants will determine the affective tone as well as the motivational nature of relief. As such, IRMO may help to understand existing variability in empirical evidence on affective and motivational underpinnings of relief. The reviewed evidence provides first support for the viablity of the process assumptions outlined in IRMO. At the same time, this evidence often fails to experimentally manipulate the theoretically important variables. Instead, our conclusions were typically based on cross-experiment comparisons. Therefore, conducting direct tests of the moderator predictions generated by IRMO is a desirable goal for future research. This may lead to some confirmations and possibly some disconfirmations of predictions generated by IRMO. In any case, we believe that such an edeavor will better our understanding of relief.

## Conflict of Interest Statement

The authors declare that the research was conducted in the absence of any commercial or financial relationships that could be construed as a potential conflict of interest.

## Footnotes

1 We use the logical disjunction to express that one of the two or both might be present.

2 In **Figure 2**, this will result in a negative difference in probabilities and thus an inhibitory influence on negative affect.

3 Although the probability difference is negative in the case of controllability, the negative correlation attached to the path from controllability assessment to behavior in **Figure 2** results in an increase in activation.

4 Following other approaches (Carver and Harmon-Jones, 2009), we refrain from further distinguishing facets of approach-avoidance motivation, such as moving toward vs. away or appetitive vs. aversive.

## References

[B1] Adams L. Faseur T. Geuens M. (2011). The influence of the self-regulatory focus on the effectiveness of stop-smoking campaigns for young smokers. *J. Consum. Aff.* 45 275–305 10.1111/j.1745-6606.2011.01203.x

[B2] Amsel A. Maltzman I. (1950). The effect upon generalized drive strength of emotionality as inferred from the level of consummatory response. *J. Exp. Psychol.* 40 563–569 10.1037/h0061101 14784545

[B3] Andreatta M. Fendt M. Mühlberger A. Wieser M. J. Imobersteg S. Yarali A. (2012). Onset and offset of aversive events establish distinct memories requiring fear- and reward network. *Learn. Mem.* 19 518–526 10.1101/lm.026864.112 23073641

[B4] Andreatta M. Mühlberger A. Glotzbach-Schoon E. Pauli P. (2013). Pain predictability reverses valence ratings of a relief-associated stimulus. *Front. Syst. Neurosci.* 7:53 10.3389/fnsys.2013.00053 PMC3782145 24068989

[B5] Andreatta M. Mühlberger A. Yarali A. Gerber B. Pauli P. (2010). A rift between implicit and explicit conditioned valence in human pain relief learning. *Proc. R. Soc. B Biol. Sci.* 277 2411–2416 10.1098/rspb20100103 PMC2894900 20356893

[B6] Arcediano F. Ortega N. Matute H. (1996). A behavioural preparation for the study of human Pavlovian conditioning. *Q. J. Exp. Psychol. B* 49 270–283. 8828400 10.1080/713932633

[B7] Baas J. M. P. Kenemans J. L. Böcker K. B. E. Verbaten M. N. (2002). Threat-induced cortical processing and startle potentiation. *Neuroreport* 13 133–137 10.1097/00001756-200201210-00031 11926166

[B8] Baas M. De Dreu C. K. W. Nijstad B. A. (2011). When prevention promotes creativity: the role of mood, regulatory focus, and regulatory closure. *J. Pers. Soc. Psychol.* 100 794–809 10.1037/a0022981 21381857

[B9] Baker T. B. Piper M. E. McCarthy D. E. Majeskie M. R. Fiore M. C. (2004). Addiction motivation reformulated: an affective processing model of negative reinforcement. *Psychol. Rev.* 111 33–51 10.1037/0033-295X.111.1.33 14756584

[B10] Bastian B. Jetten J. Hornsey M. J. Leknes S. (2014). The positive consequences of pain: a biopsychosocial approach. *Pers. Soc. Psychol. Rev.* 18 256–279 10.1177/1088868314527831 24727972

[B11] Benning S. D. Patrick C. J. Lang A. R. (2004). Emotional modulation of the post- auricular reflex. *Psychophysiology* 41 426–432 10.1111/j.1469-8986.00160.x 15102128

[B12] Berger D. F. Brush F. R. (1975). Rapid acquisition of discrete-trial lever-press avoidance: effects of signal-shock interval. *J. Exp. Anal. Behav.* 24 227–239 10.1901/jeab.1975.24-227 16811875 PMC1333403

[B13] Berger D. F. Starzec J. J. (1988). Contrasting lever-press avoidance behaviors of spontaneously hypertensive and normotensive rats (*Rattus norvegicus*). *J. Comp. Psychol.* 102 279–286 10.1037/0735-7036.102.3.279 3180735

[B14] Berkowitz L. (1989). Frustration aggression hypothesis – Examination and reformulation. *Psychol. Bull.* 106 59–73 10.1037/0033-2909.106.1.59 2667009

[B15] Blanco F. Matute H. Vadillo M. (2011). Making the uncontrollable seem controllable: the role of action in the illusion of control. *Q. J. Exp. Psychol.* 64 1290–1304 10.1080/17470218.2011.552727 21432736

[B16] Bossuyt E. Moors A. De Houwer J. (2014). On angry approach and fearful avoidance: the goal-dependent nature of emotional approach and avoidance tendencies. *J. Exp. Soc. Psychol.* 50 118–124 10.1016/j.jesp.2013.09.009

[B17] Bresin K. Gordon K. H. (2013). Changes in negative affect following pain (vs. nonpainful) stimulation in individuals with and without a history of nonsuicidal self-injury. *Personal. Disord.* 4 62–66 10.1037/a0025736 22452770

[B18] Bresin K. Gordon K. H. Bender T. W. Gordon L. J. Joiner T. E. Jr. (2010). No pain, no change: reductions in prior negative affect following physical pain. *Motiv. and Emotion* 34 280–287. 10.1007/s11031-010-9168-7

[B19] Bromage B. K. Scavio M. J. (1978). Effects of an aversive CS^+^ and CS^-^ under deprivation upon successive classical appetitive and aversive conditioning. *Anim. Learn. Behav.* 6 57–65 10.3758/BF03212003

[B20] Cándido A. González F. de Brugada I. (2004). Safety signals from avoidance learning but not from yoked classical conditioning training pass both summation and retardation tests for inhibition. *Behav. Process.* 66 153–160 10.1016/j.beproc.2004.01.011 15110917

[B21] Carlezon W. A. J. Thomas M. J. (2009). Biological substrates of reward and aversion: a nucleus accumbens activity hypothesis. *Neuropharmacology* 56 122–132 10.1016/j.neuropharm.2008.06.075 18675281 PMC2635333

[B22] Carver C. S. (2001). Affect and the functional bases of behavior: on the dimensional structure of affective experience. *Pers. Soc. Psychol. Rev.* 5 345–356 10.1207/S15327957PSPR0504_4

[B23] Carver C. S. (2004). Negative affects deriving from the behavioral approach system. *Emotion* 4 3–22 10.1037/1528-3542.4.1.3 15053723

[B24] Carver C. S. (2009). Threat sensitivity, incentive sensitivity, and the experience of relief. *J. Pers.* 77 125–138 10.1111/j.1467-6494.2008.00540.x 19076994

[B25] Carver C. S. Harmon-Jones E. (2009). Anger is an approach-related affect: evidence and implications. *Psychol. Bull.* 135 183–204 10.1037/a0013965 19254075

[B26] Carver C. S. Scheier M. F. (1990). Origins and functions of positive and negative affect: a control process view. *Psychol. Rev.* 97 19–35 10.1037/0033-295X.97.1.19

[B27] Carver C. S. Scheier M. F. (1998). *On the Self-Regulation of Behavior.* New York, NY: Cambridge University Press. 10.1017/CBO9781139174794

[B28] Carver C. S. Scheier M. F. (2002). Control processes and self-organization as complementary principles underlying behavior. *Pers. Soc. Psychol. Rev.* 6 304–315 10.1207/S15327957PSPR0604_05

[B29] Carver C. S. Scheier M. F. (2011). “Self-regulation of action and affect,” in *Handbook of Self-Regulation: Research, Theory, and Applications*, 2nd Edn eds Vohs K. D. Baumeister R. F. (New York, NY: Guilford Press), 3–21.

[B30] Cole R. P. Miller R. R. (1999). Conditioned excitation and conditioned inhibition acquired through backward conditioning. *Learn. Motiv.* 30 129–156 10.1006/lmot.1998.1027

[B31] Cook M. Mineka S. Trumble D. (1987). The role of response-produced and exteroceptive feedback in the attenuation of fear over the course of avoidance learning. *J. Exp. Psychol. Anim. Behav. Process.* 13 239–249. 10.1037/0097-7403.13.3.239

[B32] Davis H. Memmott J. Hurwitz H. M. (1976). Effects of signals preceding and following shock on baseline responding during a conditioned-suppression procedure. *J. Exp. Anal. Behav.* 25 263–277 10.1901/jeab.1976.25-263 16811910 PMC1333460

[B33] Delgado M. R. Jou R. L. LeDoux J. E. Phelps E. A. (2009). Avoiding negative outcomes: tracking the mechanisms of avoidance learning in humans during fear conditioning. *Front. Behav. Neurosci.* 3:33 10.3389/neuro.08.033.2009 PMC2762377 19847311

[B34] DeVito P. L. Fowler H. (1994). Positive and negative transfer of conditioned aversive stimuli to a conditioned appetitive excitor as a function of aversive US intensity. *Anim. Learn. Behav.* 22 195–202 10.3758/BF03199920

[B35] Dinsmoor J. A. (2001). Stimuli inevitably generated by behavior that avoids electric shock are inherently reinforcing. *J. Exp. Anal. Behav.* 75 311–333 10.1901/jeab.2001.75-311 11453621 PMC1284820

[B36] Dinsmoor J. A. Sears G. W. (1973). Control of avoidance by a response-produced stimulus. *Learn. Motiv.* 4 284–293 10.1016/0023-9690(73)90018-0

[B37] Dolinski D. Ciszek M. Godlewski K. Zawadzki M. (2002). Fear-then-relief, mindlessness, and cognitive deficits. *Eur. J. Soc. Psychol.* 32 435–447 10.1002/ejsp.100

[B38] Dolinski D. Nawrat R. (1998). “Fear-then-relief” procedure for producing compliance: beware when the danger is over. *J. Exp. Soc. Psychol.* 34 27–50 10.1006/jesp.1997.1341

[B39] Dollard J. Doob L. Miller N. Mowrer O. Sears R. (1939). *Frustration and Aggression.* New Haven, CT: Yale University Press. 10.1037/10022-000

[B40] Eder A. B. Dignath D. (2014). I like to get nothing: implicit and explicit evaluation of avoided negative outcomes. *J. Exp. Psychol. Anim. Learn. Cogn.* 40 55–62 10.1037/xan0000005 24893108

[B41] Ellsworth P. C. Smith C. A. (1988). Shades of joy: patterns of appraisal differentiating pleasant emotions. *Cogn. Emot.* 2 301–331 10.1080/02699938808412702

[B42] Faddegon K. Scheepers D. Ellemers N. (2008). If we have the will, there will be a way: regulatory focus as a group identity. *Eur. J. Soc. Psychol.* 38 880–895 10.1002/ejsp.483

[B43] Falls W. A. Davis M. (1995). Lesions of the central nucleus of the amygdala block conditioned excitation, but not conditioned inhibition of fear as measured with the fear-potentiated startle effect. *Behav. Neurosci.* 109 379–387 10.1037/0735-7044.109.3.379 7662148

[B44] Falomir-Pichastor J. M. Mugny G. Gabarrot F. Quiamzade A. (2011). A regulatory fit perspective in majority versus minority support to attitudes toward homosexuals. *Group Process. Intergroup Relat.* 14 45–62 10.1177/1368430210376077

[B45] Falomir-Pichastor J. M. Mugny G. Quiamzade A. Gabarrot F. (2008). Motivations underlying attitudes: regulatory focus and majority versus minority support. *Eur. J. Soc. Psychol.* 38 587–600 10.1002/ejsp.494

[B46] Favazza A. R. (1998). The coming of age of self-mutilation. *J. Nerv. Ment. Dis.* 186 259–268 10.1097/00005053-199805000-00001 9612442

[B47] Fernando A. B. P. Urcelay G. P. Mar A. C. Dickinson A. Robbins T. W. (2013). Comparison of the conditioned reinforcing properties of a safety signal and appetitive stimulus: effects of d-amphetamine and anxiolytics. *Psychopharmacology (Berl.)* 227 195–208 10.1007/s00213-012-2952-1 23299096 PMC3636441

[B48] Förster J. Liberman N. Friedman R. (2007). Seven principles of goal activation: a systematic approach to distinguishing goal priming from priming of non-goal constructs. *Pers. Soc. Psychol. Rev.* 11 211–233 10.1177/1088868307303029 18453462

[B49] Franklin J. C. Hessel E. T. Aaron R. V. Arthur M. S. Heilbron N. Prinstein M. J. (2010). The functions of nonsuicidal self-injury: support for cognitive–affective regulation and opponent processes from a novel psychophysiological paradigm. *J. Abnorm. Psychol.* 119 850–862 10.1037/a0020896 20939652 PMC4163759

[B50] Franklin J. C. Lee K. M. Hanna E. K. Prinstein M. J. (2013a). Feeling worse to feel better: pain-offset relief simultaneously stimulates positive affect and reduces negative affect. *Psychol. Sci.* 24 521–529 10.1177/0956797612458805 23459871

[B51] Franklin J. C. Puzia M. E. Lee K. M. Lee G. E. Hanna E. K. Spring V. L. (2013b). The nature of pain offset relief in nonsuicidal self-injury: a laboratory study. *Clin. Psychol. Sci.* 1 110–119 10.1177/2167702612474440

[B52] Frijda N. H. Kuipers P. ter Schure E. (1989). Relations among emotion, appraisal, and emotional action readiness. *J. Pers. Soc. Psychol.* 57 212–228 10.1037/0022-3514.57.2.212

[B53] Fujiwara J. Tobler P. N. Taira M. Iijima T. Tsutsui K.-I. (2009). A parametric relief signal in human ventrolateral prefrontal cortex. *Neuroimage* 44 1163–1170 10.1016/j.neuroimage.2008.09.050 18992349

[B54] Gable P. A. Harmon-Jones E. (2009). Postauricular reflex responses to pictures varying in valence and arousal. *Psychophysiology* 46 487–490 10.1111/j.1469-8986.2009.00794.x 19226306

[B55] Genud-Gabai R. Klavir O. Paz R. (2013). Safety signals in the primate amygdala. *J. Neurosci.* 33 17986–17994 10.1523/JNEUROSCI.1539-13.2013 24227710 PMC3828455

[B56] Gerber B. Yarali A. Diegelmann S. Wotjak C. T. Pauli P. Fendt M. (2014). Pain-relief learning in flies, rats, and man: basic research and applied perspectives. *Learn. Mem.* 21 232–252 10.1101/lm.032995.113 24643725 PMC3966540

[B57] Gilovich T. Griffin D. Kahneman D. (2002). “The psychology of intuitive judgment,” in *Heuristics and Biases*, eds Gilovich T. Griffin D. Kahneman D. (New York, NY: Cambridge University Press). 10.1017/CBO9780511808098

[B58] Gray J. A. (1971). *The Psychology of Fear and Stress.* New York, NY: Mcgraw-Hill Book Company.

[B59] Gray J. A. (1987). *The Psychology of Fear and Stress*, 2nd Edn. New York, NY: Cambridge University Press.

[B60] Gray J. A. McNaughton N. (2000). *The Neuropsychology of Anxiety.* New York: Oxford University Press.

[B61] Grelle M. J. James J. H. (1981). Conditioned Inhibition of Fear: evidence for a Competing Response Mechanism. *Learn. Motiv.* 12 300–320 10.1016/0023-9690(81)90011-4

[B62] Hamm A. O. Greenwald M. K. Bradley M. M. Lang P. J. (1993). Emotional learning, hedonic change, and the startle probe. *J. Abnorm. Psychol.* 102 453–465 10.1037/0021-843X.102.3.453 8408958

[B63] Hammond L. J. (1966). Increased responding to CS- in differential CER. *Psychon. Sci.* 5 337–338 10.3758/BF03328427

[B64] Haraway M. M. Maples E. G. Cooper S. C. (1984). Contiguous approach conditioning: a model for Sidman avoidance learning. *Psychol. Rep.* 55 291–295 10.2466/pr0.1984.55.1.291

[B65] Harmon-Jones E. Sigelman J. D. Bohlig A. Harmon-Jones C. (2003). Anger, coping, and frontal cortical activity: the effect of coping potential on anger-induced left frontal activity. *Cogn. Emot.* 17 1–24 10.1080/02699930302278 29715737

[B66] Higgins E. T. (1987). Self-discrepancy: a theory relating self and affect. *Psychol. Rev.* 94 319–340 10.1037/0033-295X.94.3.319 3615707

[B67] Higgins E. T. (1996). “Emotional experiences: the pains and pleasures of distinct regulatory systems,” in *Emotion: Interdisciplinary Perspectives*, eds Kavanaugh R. D. Zimmerberg B. Fein S.(Hillsdale, NJ: Lawrence Erlbaum Associates, Inc.), 203–241.

[B68] Higgins E. T. (1997). Beyond pleasure and pain. *Am. Psychol.* 52 1280–1300 10.1037/0003-066X.52.12.1280 9414606

[B69] Higgins E. T. (2001). “Promotion and prevention experiences: relating emotions to nonemotional motivational states,” in *Handbook of Affect and Social Cognition*, ed.Forgas J. P. (Mahwah, NJ: Lawrence Erlbaum Associates Publishers), 186–211.

[B70] Higgins E. T. Shah J. Friedman R. (1997). Emotional responses to goal attainment: strength of regulatory focus as moderator. *J. Pers. Soc. Psychol.* 72:515 10.1037/0022-3514.72.3.515 9120782

[B71] Higgins T. Tykocinski O. (1992). Self-discrepancies and biographical memory: personality and cognition at the level of psychological situation. *Pers. Soc. Psychol. Bull.* 18 527–535 10.1177/0146167292185002

[B72] Hoffman H. S. Fleshler M. (1964). Stimulus aspects of aversive controls: stimulus generalization of conditioned suppression following discrimination training. *J. Exp. Anal. Behav.* 7 233–239 10.1901/jeab.1964.7-233 14143911 PMC1404411

[B73] Holmes N. M. Marchand A. R. Coutureau E. (2010). Pavlovian to instrumental transfer: a neurobehavioural perspective. *Neurosci. Biobehav. Rev.* 34 1277–1295 10.1016/j.neubiorev.2010.03.007 20385164

[B74] Hommel B. (2010). “Grounding attention in action control: the intentional control of selection,” in *Effortless Attention: A New Perspective in the Cognitive Science of Attention and Action* ed. Bruya B. (Cambridge, MA: The MIT Press) 121–140 10.7551/mitpress/9780262013840.003.0006

[B75] Idson L. C. Liberman N. Higgins E. T. (2000). Distinguishing gains from nonlosses and losses from nongains: a regulatory focus perspective on hedonic intensity. *J. Exp. Soc. Psychol.* 36 252–274 10.1006/jesp.1999.1402

[B76] Jensen J. McIntosh A. Crawley A. Mikulis D. Remington G. Kapur S. (2003). Direct activation of the ventral striatum in anticipation of aversive stimuli. *Neuron* 40 1251–1257 10.1016/S0896-6273(03)00724-4 14687557

[B77] Jensen J. Smith A. J. Willeit M. Crawley A. P. Mikulis D. J. Vitcu I. (2007). Separate brain regions code for salience vs. valence during reward prediction in humans. *Hum. Brain Mapp.* 28 294–302 10.1002/hbm.20274 16779798 PMC6871333

[B78] Josselyn S. A. Falls W. A. Gewirtz J. C. Pistell P. Davis M. (2005). The nucleus accumbens is not critically involved in mediating the effects of a safety signal on behavior. *Neuropsychopharmacology* 30 17–26 10.1038/sj.npp.1300530 15257308

[B79] Jovanovic T. Norrholm S. D. Keyes M. Fiallos A. Jovanovic S. Myers K. M. (2006). Contingency awareness and fear inhibition in a human fear-potentiated startle paradigm. *Behav. Neurosci.* 120 995–1004 10.1037/0735-7044.120.5.995 17014251 PMC3740393

[B80] Kim H. Shimojo S. O’Doherty J. P. (2006). Is avoiding an aversive outcome rewarding? Neural substrates of avoidance learning in the human brain. *PLoS Biol.* 4:e233 10.1371/journal.pbio.0040233 PMC1484497 16802856

[B81] Kinsman R. A. Bixenstine V. E. (1968). Secondary reinforcement and shock termination. *J. Exp. Psychol.* 76 62–68 10.1037/h0025308

[B82] Kohls G. Perino M. T. Taylor J. M. Madva E. N. Cayless S. J. Troiani V. (2013). The nucleus accumbens is involved in both the pursuit of social reward and the avoidance of social punishment. *Neuropsychologia* 51 2062–2069 10.1016/j.neuropsychologia.2013.07.020 23911778 PMC3799969

[B83] Krank M. D. (1985). Asymmetrical effects of Pavlovian excitatory and inhibitory aversive transfer on Pavlovian appetitive responding and acquisition. *Learn. Motiv.* 16 35–62 10.1016/0023-9690(85)90003-7

[B84] Krieglmeyer R. De Houwer J. Deutsch R. (2013). On the nature of automatically triggered approach–avoidance behavior. *Emot. Rev.* 5 280–284 10.1177/1754073913477501

[B85] Krieglmeyer R. Deutsch R. (2013). Approach does not equal approach: angry facial expressions evoke approach only when it serves aggression. *Soc. Psychol. Personal. Sci.* 4 607–614 10.1177/1948550612471060

[B86] Lang P. J. Bradley M. M. Cuthbert B. N. (1990). Emotion, attention, and the startle reflex. *Psychol. Rev.* 97 377–395 10.1037/0033-295X.97.3.377 2200076

[B87] Lang P. J. Bradley M. M. Cuthbert B. N. (1992). A motivational analysis of emotion: reflex-cortex connections. *Psychol. Sci.* 3 44–49 10.1111/j.1467-9280.1992.tb00255.x

[B88] Langer E. J. (1975). The illusion of control. *J. Pers. Soc. Psychol.* 32 311–328 10.1037/0022-3514.32.2.311

[B89] Langer E. J. Roth J. (1975). Heads I win, tails it’s chance: the illusion of control as a function of the sequence of outcomes in a purely chance task. *J. Pers. Soc. Psychol.* 32 951–955 10.1037/0022-3514.32.6.951

[B90] Lawrence J. W. Carver C. S. Scheier M. F. (2002). Velocity toward goal attainment in immediate experience as a determinant of affect. *J. Appl. Soc. Psychol.* 32 788–802 10.1111/j.1559-1816.2002.tb00242.x

[B91] Lazarus R. S. (1991). *Emotion and Adaptation.* New York, NY: Oxford University Press.

[B92] Leknes S. Berna C. Lee M. C. Snyder G. D. Biele G. Tracey I. (2013). The importance of context: when relative relief renders pain pleasant. *Pain* 154 402–410 10.1016/j.pain.2012.11.018 23352758 PMC3590449

[B93] Leknes S. Brooks J. C. W. Wiech K. Tracey I. (2008). Pain relief as an opponent process: A psychophysical investigation. *Eur. J. Neurosci.* 28 794–801 10.1111/j.1460-9568.2008.06380.x 18671736

[B94] Leknes S. Lee M. C. Berna C. Andersson J. Tracey I. (2011). Relief as a reward: hedonic and neural responses to safety from pain. *PLoS ONE* 6:e17870 10.1371/journal.pone.0017870 PMC3072382 21490964

[B95] Lench H. C. Flores S. A. Bench S. W. (2011). Discrete emotions predict changes in cognition, judgment, experience, behavior, and physiology: a meta-analysis of experimental emotion elicitations. *Psychol. Bull.* 137 834–855 10.1037/a0024244 21766999

[B96] Leone L. Perugini M. Bagozzi R. P. (2005). Emotions and decision making: regulatory focus moderates the influence of anticipated emotions on action evaluations. *Cogn. Emot.* 19 1175–1198 10.1080/02699930500203203

[B97] Levita L. Hare T. A. Voss H. U. Glover G. Ballon D. J. Casey B. J. (2009). The bivalent side of the nucleus accumbens. *Neuroimage* 44 1178–1187 10.1016/j.neuroimage.2008.09.039 18976715 PMC2659952

[B98] Levita L. Hoskin R. Champi S. (2012). Avoidance of harm and anxiety: a role for the nucleus accumbens. *Neuroimage* 62 189–198 10.1016/j.neuroimage.2012.04.059 22569544

[B99] Lipp O. V. Siddle D. A. T. Dall P. J. (2003). The effects of unconditional stimulus valence and conditioning paradigm on verbal, skeleto-motor and autonomic indices of human Pavlovian conditioning. *Learn. Motiv.* 34 32–51 10.1016/S0023-9690(02)00507-6

[B100] Lohr J. M. Olatunji B. O. Sawchuk C. N. (2007). A functional analysis of danger and safety signals in anxiety disorders. *Clin. Psychol. Rev.* 27 114–126 10.1016/j.cpr.2006.07.005 16997437

[B101] Mallan K. M. Lipp O. V. (2007). Does emotion modulate the blink reflex in human conditioning? Startle potentiation during pleasant and unpleasant cues in the picture-picture paradigm. *Psychophysiology* 44 737–748 10.1111/j.1469-8986.2007.00541.x 17532801

[B102] McKay-Nesbitt J. Bhatnagar N. Smith M. C. (2013). Regulatory fit effects of gender and marketing message content. *J. Bus. Res.* 66 2245–2251 10.1016/j.jbusres.2012.02.004

[B103] Migo E. M. Corbett K. Graham J. Smith S. Tate S. Moran P. M. (2006). A novel test of conditioned inhibition correlates with personality measures of schizotypy and reward sensitivity. *Behav. Brain Res.* 168 299–306 10.1016/j.bbr.2005.11.021 16386317

[B104] Moscovitch A. LoLordo V. M. (1968). Role of safety in the Pavlovian backward fear conditioning procedure. *J. Comp. Physiol. Psychol.* 66 673–678 10.1037/h0026548 5721495

[B105] Mowrer O. H. (1960). *Learning Theory and Behavior.* New York: Wiley. 10.1037/10802-000

[B106] Murray A. K. Strandberg J. M. (1965). Development of a conditioned positive reinforcer through removal of an aversive stimulus. *J. Comp. Physiol. Psychol.* 60 281–283 10.1037/h0022363 5832362

[B107] Navratilova E. Porreca F. (2014). Reward and motivation in pain and pain relief. *Nat. Neurosci.* 17 1304–1312 10.1038/nn.3811 25254980 PMC4301417

[B108] Navratilova E. Xie J. Y. Okuna A. Qu C. Eyde N. Ci S. (2012). Pain relief produces negative reinforcement through activation of mesolimbic reward –valuation circuitry. *Proc. Natl. Acad. Sci. U.S.A.* 109 20709–20713 10.1073/pnas.1214605109 23184995 PMC3528534

[B109] Neumann R. Förster J. Strack F. (2003). “Motor compatibility: the bidirectional link between behavior and evaluation,” in *The Psychology of Evaluation: Affective Processes in Cognition and Emotion*, eds Musch J. Klauer K. C. (Mahwah, NJ:Lawrence Erlbaum Associates Publishers) 371–391.

[B110] Niznikiewicz M. A. Delgado M. R. (2011). Two sides of the same coin: learning via positive and negative reinforcers in the human striatum. *Dev. Cogn. Neurosci.* 1 494–505 10.1016/j.dcn.2011.07.006 21922033 PMC3171207

[B111] Ortony A. Clore G. L. Collins A. (1988). *The Cognitive Structure of Emotions.* Cambridge: Cambridge University Press. 10.1017/CBO9780511571299

[B112] Ortony A. Turner T. J. (1990). What’s basic about basic emotions? *Psychol. Rev.* 97 315–331 10.1037/0033-295X.97.3.315 1669960

[B113] Ostafin B. D. Brooks J. J. (2011). Drinking for relief: negative affect increases automatic alcohol motivation in coping-motivated drinkers. *Motiv. Emot.* 35 285–295 10.1007/s11031-010-9194-5

[B114] Papini M. R. Dudley R. T. (1997). Consequences of surprising reward omissions. *Rev. Gen. Psychol.* 1 175–197 10.1037/1089-2680.1.2.175

[B115] Pekrun R. Goetz T. Titz W. Perry R. P. (2002). Academic emotions in students’ self- regulated learning and achievement: a program of qualitative and quantitative research. *Educ. Psychol.* 37 91–105 10.1207/S15326985EP3702_4

[B116] Peterson C. K. Harmon-Jones E. (2012). Toward an understanding of the emotion- modulated startle eyeblink reflex: the case of anger. *Psychophysiology* 49 1509–1522 10.1111/j.1469-8986.2012.01469.x 22994146

[B117] Phaf R. H. Mohr S. E. Rotteveel M. Wicherts J. M. (2014). Approach, avoidance, and affect: a meta-analysis of approach-avoidance tendencies in manual reaction time tasks. *Front. Psychol.* 5:378 10.3389/fpsyg.2014.00378 PMC4021119 24847292

[B118] Prinz J. J. (2004). *Gut Reactions: A Perceptual Theory of Emotion*. New York, NY: Oxford University Press.

[B119] Ray O. S. Stein L. (1959). Generalization of conditioned suppression. *J. Exp. Anal. Behav.* 2 357–361 10.1901/jeab.1959.2-357 14436619 PMC1403903

[B120] Reisenzein R. (2009). Emotional experience in the computational belief–desire theory of emotion. *Emot. Rev.* 1 214–222 10.1177/1754073909103589

[B121] Reisenzein R. Spielhofer C. (1994). Subjectively salient dimensions of emotional appraisal. *Motiv. Emot.* 18 31–77 10.1007/BF02252474

[B122] Rescorla R. A. (1969). Pavlovian conditioned inhibition. *Psychol. Bull.* 72 77–94 10.1037/h0027760

[B123] Rescorla R. A. Lolordo V. M. (1965). Inhibition of avoidance behavior. *J. Comp. Physiol. Psychol.* 59 406–412 10.1037/h0022060 14313781

[B124] Rescorla R. A. Solomon R. L. (1967). Two-process learning theory: relationships between Pavlovian conditioning and instrumental learning. *Psychol. Rev.* 74 151–182 10.1037/h0024475 5342881

[B125] Riebe C. J. Pamplona F. Kamprath K. Wotjak C. T. (2012). Fear relief - toward a new conceptual frame work and what endocannabinoids gotta do with it. *Neuroscience* 204 159–185 10.1016/j.neuroscience.2011.11.057 22173015

[B126] Robinson T. E. Berridge K. C. (1993). The neural basis of drug craving: An incentive- sensitization theory of addiction. *Brain Res. Rev.* 18 247–291 10.1016/0165-0173(93)90013-P 8401595

[B127] Rogan M. T. Leon K. S. Perez D. L. Kandel E. R. (2005). Distinct neural signatures for safety and danger in the amygdala and striatum of the mouse. *Neuron* 46 309–320 10.1016/j.neuron.2005.02.017 15848808

[B128] Roseman I. J. (1984). Cognitive determinants of emotion: a structural theory. *Rev. Pers. Soc. Psychol.* 5 11–36.

[B129] Roseman I. J. (1991). Appraisal determinants of discrete emotions. *Cogn. Emot.* 5 161–200 10.1080/02699939108411034

[B130] Roseman I. J. (1996). Appraisal determinants of emotions: constructing a more accurate and comprehensive theory. *Cogn. Emot.* 10 241–278 10.1080/026999396380240

[B131] Roseman I. J. (2001). “A model of appraisal in the emotion system: integrating theory, research, and applications,” in *Appraisal Processes in Emotion: Theory, Methods, Research*, eds Scherer K. R. Schorr A. Johnstone T. (New York, NY: Oxford University Press) 68–91.

[B132] Roseman I. J. (2013). Appraisal in the emotion system: coherence in strategies for coping. *Emot. Rev.* 5 141–149 10.1177/1754073912469591

[B133] Roseman I. J. Evdokas A. (2004). Appraisals cause experienced emotions: experimental evidence. *Cogn. Emot.* 18 1–28 10.1080/02699930244000390

[B134] Roseman I. J. Spindel M. S. Jose P. E. (1990). Appraisals of emotion-eliciting events: testing a theory of discrete emotions. *J. Pers. Soc. Psychol.* 59 899–915 10.1037/0022-3514.59.5.899

[B135] Russell J. A. (2003). Core affect and the psychological construction of emotion. *Psychol. Rev.* 110 145–172 10.1037/0033-295X.110.1.145 12529060

[B136] Sangha S. Chadick J. Z. Janak P. H. (2013). Safety encoding in the basal amygdala. *J. Neurosci.* 33 3744–3751 10.1523/JNEUROSCI.3302-12.2013 23447586 PMC6619315

[B137] Savastano H. I. Cole R. P. Barnet R. C. Miller R. R. (1999). Reconsidering conditioned inhibition. *Learn. Motiv.* 30 101–127 10.1006/lmot.1998.1020

[B138] Schlund M. W. Siegle G. J. Ladouceur C. D. Silk J. S. Cataldo M. F. Forbes E. E. (2010). Nothing to fear? Neural systems supporting avoidance behavior in healthy youths. *Neuroimage* 52 710–719 10.1016/j.neuroimage.2010.04.244 20430103 PMC2892790

[B139] Schneirla T. C. (1959). “An evolutionary and developmental theory of biphasic processes underlying approach and avoidance,” in *Nebraska Symposium on Motivation*, ed.Jones M. R. (Lincoln, NE: Nebraska University Press).

[B140] Shah J. (2003). The motivational looking glass: how significant others implicitly affect goal appraisals. *J. Pers. Soc. Psychol.* 85 424–439 10.1037/0022-3514.85.3.424 14498780

[B141] Shah J. Y. Brazy P. C. Higgins E. T. (2004). Promoting us or preventing them: regulatory focus and manifestations of intergroup bias. *Pers. Soc. Psychol. Bull.* 30 433–446 10.1177/0146167203261888 15070473

[B142] Shah J. Higgins E. T. (2001). Regulatory concerns and appraisal efficiency: the general impact of promotion and prevention. *J. Pers. Soc. Psychol.* 80 693–705 10.1037/0022-3514.80.5.693 11374743

[B143] Smith C. A. Kirby L. D. (2001). “Toward delivering on the promise of appraisal theory,” in *Appraisal Processes in Emotion: Theory, Methods, Research,* eds Scherer K. R. Schorr A. Johnstone T. (New York, NY: Oxford University Press) 121–138.

[B144] Solomon R. L. (1980). The opponent-process theory of acquired motivation: The costs of pleasure and the benefits of pain. *Am. Psychol.* 35 691–712 10.1037/0003-066X.35.8.691 7416563

[B145] Strack F. Deutsch R. (2004). Reflective and impulsive determinants of social behavior. *Pers. Soc. Psychol. Rev.* 8 220–247 10.1207/s15327957pspr0803_1 15454347

[B146] Tanimoto H. Heisenberg M. Gerber B. (2004). Event timing turns punishment to reward. *Nature* 430 983–983 10.1038/430983a 15329711

[B147] Tong E. M. (2015). Differentiation of 13 positive emotions by appraisals. *Cogn. Emot.* 29 484–503 10.1080/02699931.2014.922056 24911866

[B148] Walasek G. Wêsierska M. Zieliñski K. (1995). Conditioning of fear and conditioning of safety in rats. *Acta Neurobiol. Exp.* 55 121–132. 10.55782/ane-1995-1067 7660862

[B149] Walser M. Fischer R. Goschke T. (2012). The failure of deactivating intentions: aftereffects of completed intentions in the repeated prospective memory cue paradigm. *J. Exp. Psychol. Learn. Mem. Cogn.* 38 1030–1044 10.1037/a0027000 22288817

[B150] Walser M. Goschke T. Fischer R. (2014). The difficulty of letting go: moderators of the deactivation of completed intentions. *Psychol. Res.* 78 574–583 10.1007/s00426-013-0509-5 23934576

[B151] Weike A. I. Schupp H. T. Hamm A. O. (2008). In dubio pro defensio: initial activation of conditioned fear is not cue specific. *Behav. Neurosci.* 122 685–696 10.1037/0735-7044.122.3.685 18513138

[B152] Weiss S. J. Schindler C. W. (1989). Integrating control generated by positive and negative reinforcement on an operant baseline: appetitive-aversive interactions. *Anim. Learn. Behav.* 17 433–446 10.3758/BF03205223

[B153] Weiss S. J. Thomas D. A. Weissman R. D. (1996). Combining operant-baseline-derived conditioned excitors and inhibitors from the same and different incentive classes: an investigation of appetitive–aversive interactions. *Q. J. Exp. Psychol. B.* 49 357–381 10.1080/713932635 8962540

[B154] Winterheld H. A. Simpson J. A. (2011). Seeking security or growth: a regulatory focus perspective on motivations in romantic relationships. *J. Pers. Soc. Psychol.* 101 935–954 10.1037/a0025012 21843014

[B155] Wortman C. B. Brehm J. W. (1975). “Responses to uncontrollable outcomes: an integration of reactance theory and the learned helplessness model,” in *Advances in Experimental Social Psychology*, ed.Berkowitz L. (New York, NY: Academic Press) 277–336.

[B156] Yarali A. Niewalda T. Chen Y.-C. Tanimoto H. Duerrnagel S. Gerber B. (2008). “Pain relief” learning in fruit flies. *Anim. Behav.* 76 1173–1185 10.1016/j.anbehav.2008.05.025

[B157] Yen C.-L. Chao S.-H. Lin C.-Y. (2011). Field testing of regulatory focus theory. *J. Appl. Soc. Psychol.* 41 1565–1581 10.1111/j.1559-1816.2011.00766.x

[B158] Yi S. Baumgartner H. (2008). Motivational compatibility and the role of anticipated feelings in positively valenced persuasive message framing. *Psychol. Mark.* 25 1007–1026 10.1002/mar.20250

[B159] Yi S. Baumgartner H. (2009). Regulatory focus and message framing: a test of three accounts. *Motiv. Emot.* 33 435–443 10.1007/s11031-009-9148-y

[B160] Zanna M. P. Kiesler C. A. Pilkonis P. A. (1970). Positive and negative attitudinal affect established by classical conditioning. *J. Pers. Soc. Psychol.* 14 321–328 10.1037/h0028991 5434867

[B161] Zvolensky M. J. Lejuez C. W. Eifert G. H. (2000). Prediction and control: operational definitions for the experimental analysis of anxiety. *Behav. Res. Ther.* 38 653–663 10.1016/S0005-7967(99)00090-X 10875188

